# Bounding the execution time of parallel applications on unrelated multiprocessors

**DOI:** 10.1007/s11241-021-09375-2

**Published:** 2021-10-21

**Authors:** Petros Voudouris, Per Stenström, Risat Pathan

**Affiliations:** Chalmers University of Technology, Göteborg, Sweden

**Keywords:** Scheduling, Heterogeneous, Unrelated, DAG, Work-conserving, Makespan

## Abstract

Heterogeneous multiprocessors can offer high performance at low energy expenditures. However, to be able to use them in hard real-time systems, timing guarantees need to be provided, and the main challenge is to determine the worst-case schedule length (also known as makespan) of an application. Previous works that estimate the makespan focus mainly on the independent-task application model or the related multiprocessor model that limits the applicability of the makespan. On the other hand, the directed acyclic graph (DAG) application model and the unrelated multiprocessor model are general and can cover most of today’s platforms and applications. In this work, we propose a simple work-conserving scheduling method of the tasks in a DAG and two new approaches to finding the makespan. A set of representative OpenMP task-based parallel applications from the BOTS benchmark suite and synthetic DAGs are used to evaluate the proposed method. Based on the empirical results, the proposed approach calculates the makespan close to the exhaustive method and with low pessimism compared to a lower bound of the actual makespan calculation.

## Introduction

There is a continuously increasing demand for computational power in hard real-time systems, such as collision avoidance and mitigation function in automotive vehicles. Such a need for higher computing performance and energy efficiency has turned the focus both in academia and industry to heterogeneous multiprocessors (Esmaeilzadeh et al. 2011; Peter Greenhalgh 2011; ARM 2011). Heterogeneous multiprocessors comprise multiple computational cores with different performance and functional characteristics. A real-time parallel application can exploit such parallel heterogeneous architectures to meet challenging performance and energy efficiency demands. However, one of the main challenges to using such an architecture in a hard real-time system is to ensure *time predictability*; one has to guarantee the timeliness of a real-time parallel application a heterogeneous platform by designing an effective scheduling algorithm and doing the offline schedulability analysis. This paper, for the first time, addresses the problem of determining the worst-case schedule length (also known as the *makespan*) of parallel application on *heterogeneous* multiprocessor platform and proposes a scheduling algorithm.

We consider a general model of the application and the processing platform, which makes the results of this paper applicable to a wide variety of applications and hardware platforms. A parallel application is modeled as a directed acyclic graph (DAG) where such a DAG has a collection of nodes, i.e., tasks and directed edges between nodes, i.e., dependencies among the tasks. The expressive power of a DAG enables us to model various applications like a collection of independent tasks (Baruah et al. 2015a) and synchronous parallel tasks (Lakshmanan et al. 2010).

We consider also a general system model—*unrelated heterogeneous multiprocessor platforms* that consist of different *processor types*. On an unrelated processing platform, a task/node $\tau ^{i}$ of a DAG may execute at a different speed than another task $\tau ^{j}$ on a processor of the same type.^1^ The task-to-processor relationship in an unrelated heterogeneous platform governs how fast a particular task executes at run time. The unrelated heterogeneous multiprocessor model is one of the most general processor models that we consider in this paper (the homogeneous and related heterogeneous multiprocessor models are special cases of the unrelated multiprocessor model).

Related works on scheduling real-time systems that consider the unrelated multiprocessor model have mainly focused on independent tasks (Andersson and Raravi 2014; Chwa et al. 2015; Andersson and Raravi 2016; Baruah et al. 2019) with no dependencies and typed DAGs (Yang et al. 2016; Han et al. 2019). Earlier works that consider a DAG as the application model have focused mainly on the related multiprocessor model (Bender and Rabin 2000; Jiang et al. 2017). Our research bridges the gap between the work on the related and the unrelated models by considering both a general application model (DAGs) and a general processor model.

Scheduling algorithms play the central role in guaranteeing time predictability, i.e., computing the makespan of parallel applications. Due to the specific speed relationship that a task of the DAG has with a particular processor type, one of the main challenges is to design an effective scheduling algorithm that can well exploit all the computational units of a heterogeneous parallel architecture. A second challenge is to perform offline schedulability analysis by considering the execution of the tasks under the scheduling algorithm so that a safe and tight upper bound on the worst-case schedule length (makespan) can be computed. Such a makespan can be used, for example, to determine whether the deadline of an application will be met or not when the system is actually put in mission.

Many of the well-known schedulability analysis techniques for homogeneous multiprocessors cannot be trivially applied to heterogeneous multiprocessors (Gupta et al. 2012). One of the fundamental problems is the presence of *timing anomalies* (Graham 1969). Note that a timing anomaly is already known to exist for the homogeneous multiprocessor model, which is a special case of the unrelated multiprocessor model (Voudouris et al. 2017; Pathan et al. 2018; Chen et al. 2019). Therefore, an example of a DAG—similar to that of (Voudouris et al. 2017) can also be constructed to demonstrate the presence of timing anomalies in the unrelated multiprocessor model. A method to avoid such anomalies for homogeneous multiprocessors is to preserve strictly, also at run-time, the order of *start time of the execution of the tasks* that was determined at analysis time (Voudouris et al. 2017; Pathan et al. 2018; Chen et al. 2019). Unfortunately, enforcing such an order of starting the tasks’ execution is not enough to guarantee anomaly-freedom on unrelated machines because of the different speed relationships that each task has with each processor type.

This paper proposes a scheduling algorithm called the Greedy scheduler for unrelated HEterogeneous platform ($\mathcal {GHE}$) that can schedule the tasks of a DAG on an unrelated heterogeneous platform. One of the salient features of $\mathcal {GHE}$ is that it is *work-conserving* (a.k.a. greedy) (Graham 1969; Brent 1974; Blumofe and Leiserson 1999; Melani et al. 2015; Jiang et al. 2017) meaning that it always dispatches an available task whenever there is an idle processor. The scheduler $\mathcal {GHE}$ is also very general in the sense that it does not assume any specific policy like fixed or dynamic priority-based scheduling used in the literature. Since many of the fixed- and dynamic-priority-based scheduling algorithms are also work-conserving, the analysis of this paper is also applicable for such schedulers. Another facet of $\mathcal {GHE}$ is that it allows the migration of a task to some other processor to execute it at a higher speed. It will be evident later that the fact that the scheduler is work-conserving and the migratory nature of $\mathcal {GHE}$ allows us to formally derive the makespan of a parallel application and prove its correctness.

A rigorous formal analysis is conducted in this paper to tackle and understand the complex relationships the tasks of a DAG have with the unrelated processors’ types. Two different approaches—namely Comb and Fast —are proposed to determine in two different ways the makespan of a DAG executing on an unrelated heterogeneous multiprocessor under the $\mathcal {GHE}$ scheduler. The two approaches Comb and Fast mainly differ in terms of making the tradeoff between the computational complexity and tightness of the computed makespan. The first approach, Comb, is based on an exhaustive search by considering *all* the possible ways the tasks of a DAG may execute on different processors. On the other hand, the second approach, Fast, is based on considering *one* pessimistic worst-case regarding how the tasks can execute on the processors. The Comb approach computes a tighter makespan in comparison to that of using the Fast approach, but Comb has exponential time complexity while Fast can find the makespan in polynomial time.

To evaluate the proposed approaches, Fast, and Comb, we use real-world parallel applications from the BOTS benchmark suite (Duran et al. 2002) as well as randomly generated synthetic DAGs. We also compare the proposed approaches to similar work in the literature for homogeneous (Graham 1969), related (Jiang et al. 2017) multiprocessors, and typed DAGs (Han et al. 2019) to demonstrate how much we pay for using more generalized models of the application, hardware, and the scheduler with respect to that of state-of-the-art. One of the major findings we have from this empirical study is that the makespan computed using the efficient Fast approach is very close to that computed using the Comb, i.e., our analysis using the polynomial-time approach does not significantly compromise the tightness of the computed makespan. To this end, this paper makes the following contributions:

- This paper considers a general application model using DAGs, and a general hardware model for unrelated machines, to propose a general work-conserving scheduler $\mathcal {GHE}$. Consideration of such general models makes the results of this paper widely applicable to a variety of real-time systems.
- **Comb:** An exhaustive search-based approach Comb is proposed to find the makespan using a high computational complexity in order to find a tight makespan. This approach is suitable for applications that have tight deadlines.
- **Fast:** In order to reduce the computational complexity to find the makespan using the exhaustive approach of Comb, this paper proposes the polynomial-time approach Fast that can be used to find the makespan for large applications with a less tight makespan.
- The experimental evaluation presents empirical results for real-world applications based on the OpenMP applications from the BOTS benchmark suite (Duran et al. 2002), which shows the applicability of our approach to practical applications. Moreover, synthetic DAGs are used to show the sensitivity of our proposed approach to different real-world parameters. The degree of tightness that Fast sacrifices to find the makespan of the OpenMP applications in polynomial time is no more than 3% that of Comb, which shows that our proposed analysis for Fast does not introduce too much pessimism.

The rest of the paper is organized as follows: Initially, Sects. 2–4 introduce the system model, the details of the proposed $\mathcal {GHE}$ scheduler, and necessary definitions for the makespan calculation. Next, Sect. 5 provides the details of our two proposed approaches to compute makespan. Then, Sect. 6 evaluates the time complexity of the proposed approaches. We then evaluate the proposed methods in Sect. 7 quantitatively. Section 8 compares our approach with related work that uses more specialized assumptions regarding the platform and application models. Section 9 presents the related work before we conclude the paper in Sect. 10.

## System model

We consider an unrelated heterogeneous multiprocessor platform with a total of $M$ processors with different types of processors. Each of the $M$ processors belongs to exactly one of the processor types. The type of processor specifies the specialty or uniqueness of the processor. For example, the big.LITTLE multiprocessor chip from ARM has two different processor types with multiple processors that belong to each such processor type (Peter Greenhalgh 2011; ARM 2011). We assume that an unrelated platform can have from one up to $M$ processor types.

A parallel application $G$ is modeled as a directed acyclic graph (DAG) such that $G=(V, E)$, where $V=\{\tau ^{1}, \ldots , \tau ^{N} \}$ is a set of $N$ nodes that designate tasks and $E \subseteq (V \times V)$ is a set of directed edges that designate dependencies among tasks. If $(\tau ^{p},\tau ^{q}) \in E$, then $\tau ^{q}$ can start its execution only after task $\tau ^{p}$ completes. Tasks with no incoming and no outgoing edges are called source (denoted as $\tau ^{src}$) and sink (denoted as $\tau ^{sink}$), respectively. We assume that there is one source node and one sink node. If the application has multiple sources or sinks nodes, we add dummy nodes (i.e., nodes without execution time) to model the application.

A task is a sequential piece of code. Each task is characterized by a set of $$M$$ worst case execution times (WCET) depending on the processor the task executes. Without loss of generality we index the processors from 1 to $$ M$$. The WCET of task $$\tau ^{i}$$ on the $$x^{th}$$ processor is denoted by $$c^{i}_{x}$$. If a task cannot execute on the $$x^{th}$$ processors, for example, due to an incompatible instruction set architecture, then $$c^{i}_{x} =\infty $$. Because the platform has a total of $$M$$ processors, each task has $$M$$ different WCETs $$c^{i}_{1}, \ldots c^{i}_{M}$$. If the $$x^{th}$$ and $$y^{th}$$ processors are of the same type, then $$c^{i}_{x} = c^{i}_{y}$$ for $$i = 1, \ldots N$$ where $$1 \le x \le M~$$ and $$1 \le y \le M$$. In other words, each task $$\tau ^{i}$$ has the same WCET on all the processors of the same type.

We define $c^{i}_{min}$ as the minimum WCET of task $\tau ^{i}$ for any of the processors in Eq. (1) as follows:

### Definition 1

Minimum WCET of $\tau ^{i}$:

1 $$\begin{aligned} c_{{min}}^{i} : = \mathop {\min }\limits _{{x = 1}}^{M} \left\{ { c_{x}^{i}}\right\} \end{aligned}$$

The *workload* of a task is the amount of computation that a task needs to complete when executing from the beginning to completion and is equal to $c^{i}_{min}$ as is given in Eq. (1). The workload of a DAG $G$ (denoted by ${\mathcal {W}}_1$) is the sum of the workloads of all the tasks in $G$ and is given as follows:

### Definition 2

Total workload of $G$:

2 $$\begin{aligned} {\mathcal {W}}_1:= \sum _{i=1}^{N} c^{i}_{min} \end{aligned}$$

A source-to-sink path or simply a path $\gamma$ in a DAG is a sequence of nodes $\gamma = (\tau ^{p}, \tau ^{p+1},$...$, \tau ^{q-1}, \tau ^{q})$, where $(\tau ^{i},\tau ^{i+1}) \in E$ such that $p \le i < q$ and $\tau ^{p}=\tau ^{src}$ and $\tau ^{q}=\tau ^{sink}$. Let $paths$ be the set of all the paths in a DAG $G$. The workload of a path $\gamma$ is the sum of the workload of the nodes on that path and is given as follows:

3 $$\begin{aligned} {\mathcal {W}}(\gamma )~ := \sum _{\tau ^{i} \in \gamma } c^{i}_{min} \end{aligned}$$

The path with the largest workload among all the paths is called the longest or critical path (denoted using $cp$ ) and is given by Eq. (4):

4 $$\begin{aligned} cp~= \arg \max _{\gamma \in paths}{{\mathcal {W}}(\gamma )~} \end{aligned}$$

The maximum workload of any path in $G$ is given in Eq. (5):

### Definition 3

The largest workload of any path in $G$:

5 $$\begin{aligned} {\mathcal {W}}_{\infty }= {\mathcal {W}}(cp)~ \end{aligned}$$

While ${\mathcal {W}}_1$ represents the workload of the entire DAG, ${\mathcal {W}}_{\infty }$ is the maximum workload of any path of the DAG. The parameters ${\mathcal {W}}_1$ and ${\mathcal {W}}_{\infty }$ can be computed in polynomial time in the representation of the DAG and capture two important characteristics of the DAG that we will use to derive the makespan using our proposed approaches Comb and Fast. Since the definition of workload considers the minimum WCET of the nodes, no DAG can finish execution earlier than ${\mathcal {W}}_{\infty }$ (i.e., a lower bound on the makespan of $G$).

**Fig. 1:**
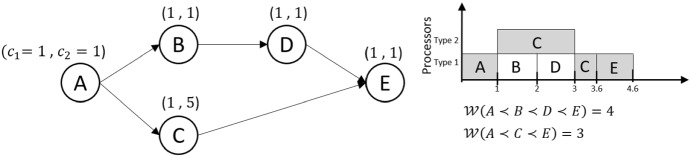
Example of a path with the largest workload $A \prec B \prec D \prec E$ that it is not the path with the longest time duration

The workload of a path is constant because it is determined by the minimum WCET among the processors of the tasks that belong to the path. However, the duration that it will take to execute a path’s workload can change from execution to execution at runtime. The WCET of the path’s tasks can be larger than their minimum WCET because, during runtime, they may be mapped to slower processors than the processors that provide the minimum WCET. Consequently, the path with the largest workload is not necessarily the path with the longest time duration to complete its execution. This situation is illustrated in Fig. 1.

The left-hand side of Fig. 1 shows a DAG. We use a platform with two processors of different processor types. Thus, each node has two different WCET. Using Eq. (3) that finds the workload of a path, the path $A \prec B \prec D \prec E$ has workload four while the path $A \prec C \prec E$ has workload three. At the right-hand side of the figure, we schedule the tasks based on their names’ lexicographic order. From this example, we can see that path $A \prec C \prec E$ determines the schedule length, which has a smaller workload than $A \prec B \prec D \prec E$. Similar examples can also be created for orders other than the lexicographic order of the tasks. To determine the makespan of a DAG, the key is to find the worst-case task-processor mapping (next section) that can occur during runtime for any execution ordering of DAG tasks. We will use the worst-case processor mapping together with the total workload and the critical path’s workload to find the makespan of a single DAG.

To model the capabilities of the unrelated processors to execute the workload of a task, we define $\delta ^{i}_{x}$ given in Eq. (6) the speed that task $\tau ^{i}$ can execute on the $x^{th}$ processor.

### Definition 4

Speed of $\tau ^{i}$ on the $x^{th}$ processor for $x=1, \ldots M~$:

6 $$\begin{aligned} \delta ^{i}_{x} := {\left\{ \begin{array}{ll} \frac{c^{i}_{min}}{c^{i}_{x}} &{} \text {if } c^{i}_{x} \ne \infty \\ 0 &{} \text {otherwise} \end{array}\right. } \end{aligned}$$

If a task $\tau ^{i}$ cannot execute on the $x^{th}$ processor, we set the speed $\delta ^{i}_{x}=0$. Note that $0 \le \delta ^{i}_{x} \le 1$ for any task $\tau ^{i}$. The smaller the WCET of $\tau ^{i}$ on a particular processor, the larger the speed that task $\tau ^{i}$ can execute on that processor is.

We define $\mathcal {O}^{i}_{y}$ as the $y^{th}$ fastest speed that task $\tau ^{i}$ can execute on some processor. For example, $\mathcal {O}^{i}_{1}$ for $y=1$ specifies the fastest speed that task $\tau ^{i}$ can execute (recall that the fastest speed is 1), $\mathcal {O}^{i}_{3}$ for $y=3$ specifies the third highest speed that task $\tau ^{i}$ can execute, and finally $\mathcal {O}^{i}_{M}$ for $y=M~$ specifies the lowest speed that task $\tau ^{i}$ can execute on some processor. It will be evident shortly that the design of $\mathcal {GHE}$ scheduler is such that it always prefers a relatively higher speed processor to execute a task. To that end, we specify the *preference for speed* of a task $\tau ^{i}$ using a sequence $\mathcal {O}^{i}_{}$ in Definition 5.

### Definition 5

Let $\mathcal {O}^{i}_{}$ be the sequence of a *non-increasing* order of speeds such that $\mathcal {O}^{i}_{} = <\mathcal {O}^{i}_{1}, \mathcal {O}^{i}_{2} \ldots \mathcal {O}^{i}_{M~}>$ where $\mathcal {O}^{i}_{y}$ is the $y^{th}$ fastest speed that task $\tau ^{i}$ can execute on a processor of the platform for $y=1, \ldots M$.

In the next section, the scheduler will use the preference for speed ($\mathcal {O}^{i}_{}$) to determine at which processor a task can execute. We will use $\mathcal {O}^{i}_{y}$ to specify the minimum preference of speed, i.e., the maximum speed, at which the task $\tau ^{i}$ can execute if all the processors that can execute task $\tau ^{i}$ with higher speeds $\mathcal {O}^{i}_{1}, \mathcal {O}^{i}_{2}, \ldots \mathcal {O}^{i}_{(y-1)}$ are busy. The $\mathcal {O}^{i}_{}$ is a key component of our approach because it allows us to determine the preference of the processor for every task. Intuitively, in contrast to homogeneous and related multiprocessors in which all the tasks have the same view of the platform (same speeds), for unrelated multiprocessors, the $\mathcal {O}^{i}_{}$ shows that every task views the platform differently because every task can have different speeds on the same processors.

## Scheduler $\mathcal {GHE}$

We present in Sect. 3.1 the details of our proposed $\mathcal {GHE}$ scheduler. An important property of $\mathcal {GHE}$, called the Greediness Property, is stated in Lemma 1. Finally, we use an example in Sect. 3.2 to illustrate the working of the scheduler using the parameters of the system model.

### Scheduler description

The $\mathcal {GHE}$ scheduler dispatches a new task awaiting execution in the ready queue when some other task finishes its execution (i.e., when some processor becomes idle). $\mathcal {GHE}$ is a work-conserving scheduler in the sense that it always dispatches a ready task if there is an idle processor. More precisely, the tasks are scheduled using $\mathcal {GHE}$ based on the next two steps: (i) Migration and (ii) Dispatching.

- **Step 1—Migration** If a processor becomes idle, the $\mathcal {GHE}$ scheduler first checks if the processor that becomes idle can execute some already executing tasks at a relatively higher speed. Without loss of generality, assume that $\tau ^{mig}$ executes on the processor to which it has been migrated at its $y^{th}$ fastest speed $\mathcal {O}^{mig}_{y}$. It is necessary for migrating $\tau ^{mig}$ that no other executing task can execute at its $k^{th}$ fastest speed for $k < y$ (please note that a lower index specifies a higher speed) on that idle processor.
- **Step 2—Dispatching** If there are tasks in the ready queue and there are idle processors, the $\mathcal {GHE}$ scheduler starts dispatching one-by-one new tasks awaiting execution in the ready queue on the fastest idle processor among all the idle processors.

The $\mathcal {GHE}$ scheduler is given in Algorithm 1. The scheduler is invoked each time some tasks finish their execution. The set of ready tasks and the indices of the processors that are idle are determined in variables readyTasksSet and idleProcSet (line 2–3), respectively. The set of tasks currently in execution is determined in variable potenMigTasksSet (line 4), and we consider these tasks for migration to an idle processor so that they can enjoy a higher speed. The set of indices of the busy processors executing the tasks in set potenMigTasksSet is determined in variable busyProcSet (line 5).

The while loop in lines 6–27 continuously checks if any of the currently executing tasks in set potenMigTasksSet can be migrated. If no such task can be migrated to any idle processor so that the task enjoys a higher speed, the while loop exits (line 24–26), and new tasks are dispatched using the second while loop in line 28–36.

The while loop in line 7 initializes the variable anyMigration to false. Line 8 initializes a set noMigTasksSet as an empty set that will be used to store the subset of the tasks of set potenMigTasksSet that are not selected for migration in the current iteration of the first while loop. The for loop in line 9–23 in each iteration considers a task $\tau ^{mig}$ from set potenMigTasksSet for migration from its current processor to an idle processor on which it would run relatively faster.

Line 10 determines the index indexMigProc of the processor such that task $\tau ^{mig}$ is executing at its $j^{th}$ highest speed on that processor with index indexMigProc. Line 12 determines the task $\tau ^{find}$ among all the potential tasks for migration from set potenMigTasksSet that can be migrated to an idle processor with index indexMigProc such that task $\tau ^{find}$ executes at the $k^{th}$ highest speed and there is no other task from set potenMigTasksSet that can execute at $h^{th}$ highest speed for some $h <k$. In other words, $\tau ^{find}$ can execute on its most preferred processor in comparison to any other task in set potenMigTasksSet.

The condition in line 14 determines if the task $\tau ^{mig}$ is the same as task $\tau ^{find}$ and $\tau ^{mig}$ can be executed at higher speed after migration, then $\tau ^{mig}$ is migrated to the processor with index indexMigProc in line 15. The set of indices of the idle processors is updated in line 16–17 and the flag anyMigration is set to true to specify that migration has occurred during the current iteration of the while loop. If the condition in line 14 is false, then task $\tau ^{mig}$ is not migrated in the current iteration of the while loop and stored in set noMigTasksSet in line 20 to consider for migration during the next iteration of the while loop. Regardless of whether migration occurs or not, task $\tau ^{mig}$ is removed from set $\texttt {potenMigTasksSet}$ and the for loop continues to consider another task from set $\texttt {potenMigTasksSet}$ for migration. In other words, the flag anyMigration is set to true if one or more tasks from set $\texttt {potenMigTasksSet}$ are selected for migration; otherwise, flag anyMigration will remain false.

When the for loop in line 9–23 completes, it is checked if migration occurred during the current iteration of the while loop or not based on the flag anyMigration. If the flag anyMigration is false, then the while loop is exited; otherwise, the while loop tries to migrate another task.

After the first while loop in line 6–27 completes, the second while loop in line 28-36 assigns the ready queue tasks to the idle processors. In line 29, an arbitrary task $\tau ^{dis}$ from set readyTasksSet is selected, and it is assigned to the idle processor on which it would run the fastest.

In line 30, the index of the processor indexNewProc is searched such that $\texttt {indexNewProc} \in \texttt {idleProcSet}$ and task $\tau ^{dis}$ executes on its $k^{th}$ fastest processor and cannot execute faster on any of the processors in set idleProcSet. Finally, we update the set of idle processors, and we remove the task from the ready queue. One by one, a new task from set readyTasksSet is dispatched to an idle processor as long as there are new tasks in the ready queue and there is at least one idle processor.

Next, we present a property, called the *Greediness Property*, of the scheduler in Lemma 1. A scheduling point is a time instant when the scheduler needs to make some new decision. Such a trivial scheduling point is at time zero. In addition, there is a scheduling point every time some task finishes its execution. We denote a time interval [*a*,*b*] a *stable time interval* such that there is no scheduling point inside the interval except at the endpoints in [*a*,*b*]. Lemma 1 proves the worst-case speeds that the tasks can execute during any stable time interval [*a*,*b*] when scheduled using the $\mathcal {GHE}$ scheduler.
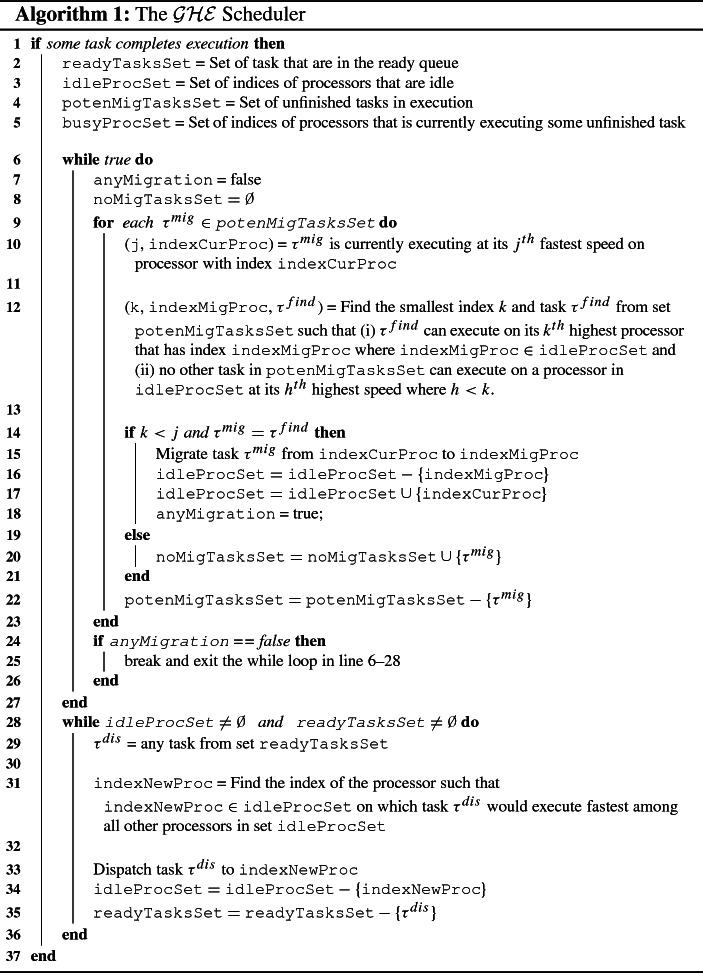


#### Lemma 1

**Greediness Property** If there are a total of *p* processors busy executing some tasks during any stable time interval under the $\mathcal {GHE}$ scheduler, then there is some task executing at least at its $k^{th}$ speed for $k=1, 2, \ldots p$ and $1 \le p \le M$.

#### Proof

Let *k*, where $0\le k \le M$, be the set of tasks that continues execution from one stable time interval to the immediately next stable time interval. Also, assume that for some *n*, where $0\le n \le M-k$, there are *n* tasks that are newly scheduled at the beginning of the next stable time interval. Let $k+n=p$, where $0\le p \le M$, be all tasks that we need to consider for execution in the new stable time interval. Let $\mathcal {O}^{*}_{x}$ denote the $x^{th}$ highest speed of some task.

We will prove this lemma considering two cases: (1) all the processors are idle, or (2) some processors are busy (i.e., some tasks from previous stable time interval continue their execution).

**Case (1)—All processors are idle** If $M$ processors are idle (i.e., $k=0$, $n=p$), then the first task that we select can be scheduled to its fastest processor that has speed one ($\mathcal {O}^{*}_{1}$) because all the processors are available. Next, the second task that we select, in the worst-case, is scheduled on its second-fastest processor ($\mathcal {O}^{*}_{2}$) because the first task may occupy the processor that provides to the second task a faster speed. Finally, the $p^{th}$ task ($\tau ^{p}$) in the worst-case is dispatched with speed $\mathcal {O}^{p}_{p}$. Because all the $p-1$ processors that provide higher speed ($\mathcal {O}^{p}_{1}$, $\mathcal {O}^{p}_{2}$, $\dots$, $\mathcal {O}^{p}_{p-1}$) for $\tau ^{p}$ may be occupied by other tasks. So for this stable time interval the *p* tasks are executing with $\mathcal {O}^{*}_{x}$, $1 \le x \le p$, respectively.

**Case (2)—Some tasks are still in execution** We separate two sub-cases: In sub-case (2.a), the tasks that are still in execution do not migrate, and in sub-case (2.b), some of the tasks that are in execution would migrate.

**Sub-case (2.a)—No migration** In this sub-case, only the new *n* tasks are scheduled on the idle processors while the *k* already-executing tasks continue executing on the processor on which they were executed in the previous stable interval. The first new task is going to execute at least with speed $\mathcal {O}^{*}_{k+1}$ because, in the worst-case, the *k* faster processors are occupied. Similarly, the remaining tasks from the *n* scheduled tasks are going to execute with speeds $\mathcal {O}^{*}_{k+2}$, $\dots$, $\mathcal {O}^{*}_{k+n}$. Therefore, the $k+n=p$ tasks are executing with speeds $\mathcal {O}^{*}_{x}$, $1 \le x \le p$ in this stable time interval.

**Sub-case (2.b)—Migration** Let $\tau ^{i}$ complete its execution at time *a* which is at the beginning of the stable time interval [*a*,*b*]. Let $\tau ^{j}$ be among the *k* tasks that still continue executing during the stable time interval [*a*,*b*]. Please recall that $\mathcal {GHE}$ first selects for migration the task that can enjoy its most preferred processor compared to other tasks. Let $\tau ^{j}$, if it migrates, has the most preferred processor among the *k* tasks that are still in execution. In the worst-case, $\tau ^{i}$ was executing before its completion on a processor that is also for $\tau ^{j}$ a faster processor. So $\tau ^{j}$ can migrate to a processor that has at least $\mathcal {O}^{j}_{k}$ speed because there are *k* tasks that can occupy the faster processors for $\tau ^{j}$. By following the $\mathcal {GHE}$ scheduler, the speeds at which tasks would start executing from the beginning of the stable interval in the worst case are $\mathcal {O}^{*}_{1}$, $\mathcal {O}^{*}_{2}$, $\dots$, $\mathcal {O}^{*}_{k}$. The *n* tasks that we need to dispatch will have the speeds^2^ in the worst-case $\mathcal {O}^{*}_{k+1}$, $\mathcal {O}^{*}_{k+2}$, $\dots$, $\mathcal {O}^{*}_{k+n}$ (follows directly from the same argument as sub-case (2.a)). Therefore, the $k+n=p$ tasks are executing with speeds $\mathcal {O}^{*}_{x}$, $1 \le x \le p$ in this stable time interval. $\square$

The main idea of the greediness property is that if $x-1$ processors are busy, then in the worst-case a task executes with its $x^{th}$ fastest speed. The scheduler selects an arbitrary task to dispatch. We prove the scheduler’s greediness without assuming any priority of the tasks. Because greediness property is oblivious to the priorities of the tasks, it holds for any priority assignment that would allow us in the next section to find a makespan computation that also holds for any priority assignment of the tasks. Finding a priority assignment that would lead to a shorter makespan is an exciting and challenging problem. However, we do not address it in this paper. In addition, we can preserve the greediness property if the scheduler is extended with preemption capability by doing the preemption before we do migration and dispatch steps because the greediness property holds for any priority assignment. Preemptive scheduling may improve the makespan, but we need to consider the preemption cost and a larger number of migrations, as we explain next.

**A note on migration** We assume that the cost of migrations is already included in the WCET of the task. The total cost of migration that a task needs to consider in the worst-case depends on the *total number* of migrations and the cost of *each* migration. Initially, in the worst-case based on the $\mathcal {GHE}$ schedule, the number of migrations for a task is bounded by $(M-1)$ because the task can migrate from its slowest processor to its fastest processor by migrating at most $(M-1)$ times. In the worst-case, a task needs to wait for $(M-2)$ other migrations by other tasks before it can migrate by considering the case where all the tasks that are in execution also need to migrate. So, in the worst case for each task we need to consider $(M-1) \cdot (M-2)$ migrations.

In case the scheduler is **preemptive**, which is equivalent to temporarily removing the tasks that are already in execution and consider them all for dispatching based on some priority order, the number of migrations that we need to consider for each task is higher compare to non-preemptive scheduling. Because when a task continues its execution after being preempted, it may be scheduled to a slower processor compared to the processor where it was executing before it was preempted. As a result for preemptive scheduling, the maximum number of migrations is $(N-1) \cdot M$. So by including the maximum number of migrations that a task may need to await before it migrates, the total number of migrations is $(N-1) \cdot M\cdot (M-2)$.

Since we can bound the maximum number of migrations, the proposed scheduler is suitable for worst-case timing analysis. Finding the cost of each migration for heterogeneous multiprocessors is a challenging problem that we do not address in this paper. The migration cost is platform-dependent, and the platform architectural characteristics are known during the WCET analysis. We assume that each migration’s cost can be computed and included within the task’s WCET.

### An example

This section presents an example of the application, platform, and scheduler using the parameters that we have defined in earlier sections. We are also going to refer to this example in later sections of this paper.

**Fig. 2:**
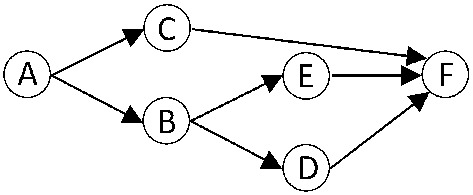
The DAG of an application with six nodes and seven dependencies

Figure 2 shows an application that we model as a DAG with six nodes A–F and seven dependencies. We assume an unrelated multiprocessor platform with two processors where each processor belongs to one unique type. The set of the WCETs of the tasks are shown in Table 1. Note that $c_{i}^{1} \ne c_{i}^{2}$ for some (in this case for all) tasks, which implies that the types of the two processors are different. There are two types of processors denoted as type 1 and type 2.

**Table 1:** The WCET of the nodes in Fig. 2 for two processors

	$c_{i}^{1}$	$c_{i}^{2}$
*A*	1	2
*B*	1	10
*C*	10	1
*D*	2	1
*E*	1	2
*F*	1	2

In Table 2, we calculate the total workload and the workload of the critical path for three cases. The column labeled as “Both type 1” is used to specify a homogeneous multiprocessor platform with two processors where both processors are of type 1. Similarly, the column labeled as “Both type 2” is used to specify a homogeneous multiprocessor platform with two processors where both processors are of type 2. Finally, the column labeled as “Unrelated: one type 1 and one type 2” is used to specify a heterogeneous multiprocessor platform with two processors where one processor is type 1, and the other is type 2.

With Eqs. (2) and (5), we calculate the total workload ${\mathcal {W}}_1$ in the second row and the workload of the critical path ${\mathcal {W}}_{\infty }$ in the third row for all the three cases. Please note that Eqs. (2) and (5) can also be applied to homogeneous multiprocessors as there is only one WCET for homogeneous multiprocessors that is equal to the minimum WCET. Eq. (7) is widely used in previous works (Graham 1969; Brent 1974; Blumofe and Leiserson 1999; Melani et al. 2015) to compute the makespan for homogeneous multiprocessors of tasks that are scheduled by a work-conserving scheduler.

7 $$\begin{aligned} T_{M(1)}~= \frac{{\mathcal {W}}_1+ (M-1) \cdot \frac{{\mathcal {W}}_{\infty }}{1} }{M} \end{aligned}$$

For the platform with two homogeneous type 1 processors, we get a makespan equal to 13.5, and for the platform with two homogeneous type 2 processors, we get a makespan equal to 17.

**Table 2:** Total workload, workload of the longest path and the makespan of the DAG are presented in Fig. 2 for two homogeneous platforms and one unrelated platform

	Both type 1	Both type 2	Unrelated: one type 1 and one type 2
${\mathcal {W}}_1$	16	18	6
${\mathcal {W}}_{\infty }$	11	16	4
Makespan	13.5	17	to be determined

In Sect. 5, we will discover the makespan with the unrelated multiprocessor platform is equal to 7.28

However, Eq. (7) cannot be trivially applied to heterogeneous multiprocessors because it does not take into account the heterogeneity of the processors. The related multiprocessors (Jiang et al. 2017) take into account the heterogeneity of the processors but assume that all the tasks can benefit equally from the architectural characteristics of the available processors, which is not realistic because they can benefit differently from the different processor types.

Before we determine the makespan for unrelated multiprocessors we first analyze the simulation of the execution of the DAG at Fig. 2 for three different scenarios.

**Fig. 3:**
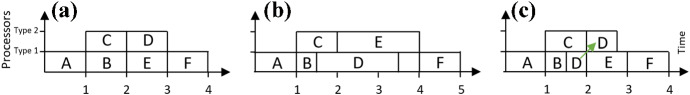
Case (**a**) presents the simulation of the execution on the unrelated platform if all the tasks execute for their WCET, case (**b**) presents the simulation if $\tau ^{B}$ completed its execution earlier without migration, and case (**c**) presents the simulation with task migration

Figure 3a presents the simulations of the execution if all the tasks execute for their WCET on the unrelated platform based on our proposed $\mathcal {GHE}$ scheduler. It can be seen that the schedule length is 4 time units. So the unrelated multiprocessor platform can execute the tasks by taking advantage of the heterogeneity of the platform and can benefit from the different architectural characteristics of the processors.

In Fig. 3b, $\tau ^{B}$ completes its execution earlier than its WCET. Since the scheduler is work-conserving, task $\tau ^{D}$ is dispatched to the processor of type 1 with speed 0.5. Similarly, $\tau ^{E}$ is dispatched to the processor of type 2 that also has the speed 0.5, and schedule length is 5. This schedule does not allow migration.

In Fig. 3c, we assume that the scheduler migrates a task to the other processor to enjoy faster speed. So, after the completion of $\tau ^{C}$, task $\tau ^{D}$ migrates to a type 2 processor to continue at a higher speed. Next, $\tau ^{E}$ is dispatched to type 1 to execute with speed one, and the schedule length is 4.

The simulations of the execution of Fig. 3a and c show the expected behavior of $\mathcal {GHE}$. However, Fig. 3b does *not* represent the expected behavior of $\mathcal {GHE}$ because between time 2 to 4 task $\tau ^{D}$ and $\tau ^{E}$ are both executing with their second-highest speed that violates the greediness property of Lemma 1. The simulation of the execution assuming that all tasks execute for their WCET cannot be used to calculate the makespan because of timing anomalies. We are trying to provide a safe upper bound of the worst-case schedule length (makespan).

## Formal tools to compute the makespan

The schedulability analysis of DAGs on an unrelated multiprocessor platform in contrast to homogeneous and related platforms cannot be done without taking into account the application. We can analyze a homogeneous platform for any DAG by knowing only the number of processors. For a related platform that has processors of different speeds, based on Funk et al. (2001), Jiang et al. (2017) we need two parameters. First, the capacity of the platform is the sum of the processors’ speeds and shows the rate that the workload of the application is executed for a given number of processors. Second, the uniformity intuitively shows how much the processors’ speed differs compared to a homogeneous platform with the same number of processors. The capacity and uniformity of a platform are fixed for any DAG for which we want to estimate the makespan. However, for an unrelated platform, the WCET of the tasks depends on the task-processor mapping; the platform characteristics can be different for every DAG.

Our approach to characterize a platform is to extend the concept of capacity and uniformity from Funk et al. (2001), Jiang et al. (2017) by taking into account the scheduler and the speeds that are derived by the task-processor mappings. Section 4.1 presents preliminary definitions and the motivation behind defining the minimum capacity and heterogeneity that we formally present in Sects. 4.2 and 4.3, respectively.

### Motivation and preliminary definitions

Computing the actual makespan of a DAG executed on unrelated multiprocessors is intractable (Garey and Johnson 2002), so we focus on computing an upper bound on the makespan. This section presents the motivation for formal analysis tools that we will use in the next section to find a safe upper bound on the makespan.

Initially, let us focus on the execution of a single task $\tau ^{i}$ and visualize its execution as rectangles. The vertical side is the speed at which the task is executing, and the horizontal side its execution time. The rectangle’s surface area shows the workload of the task given by Eq. (1).

**Fig. 4:**
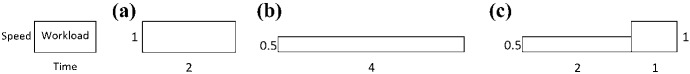
Visualization of the execution of a task $\tau ^{i}$ with workload equal to two, when (**a**) is mapped to a processor with speed 1, (**b**) with speed 0.5 and (**c**) initially with speed 0.5 and then it migrates to a processor with speed 1. Whatever is the task-processor mapping the sum of the area of rectangles for all the cases is constant and equal to the workload

Let $\tau ^{i}$ have a workload equal to two. Fig. 4 shows an abstract view of the execution of $\tau ^{i}$ for three scenarios. First, (a) shows the cases that $\tau ^{i}$ is mapped to a processor that provides to $\tau ^{i}$ speed one, so $\tau ^{i}$ completes its execution after two time units. By computing the area of the rectangle, we find the workload of $\tau ^{i}$ which is two. At (b), the $\tau ^{i}$ is mapped to a processor that offers speed 0.5 to $\tau ^{i}$ and as a result, the execution time is four, which is greater compared to (a). Again the workload is two by computing the area of the rectangle. At (c) $\tau ^{i}$ initially is mapped to a processor that offers speed 0.5 for two time units, and then it migrates to a processor that provides speed one for one time unit. The execution time for $\tau ^{i}$ for the (c) case is three time units, but again the sum of the area of the rectangles is two, which is equal to the workload of the $\tau ^{i}$.

This example illustrates that the task-processor mapping can lead to different execution times. However, we observe that the workload of the task remains constant regardless of the task-processor mapping. Based on this observation, we will inflate the DAG’s workload to bound all the schedule lengths that we can get for all the possible task-processor mappings. The inflation will introduce pessimism to the schedule length of the DAG that captures all the possible task-processor mappings in order to find a safe estimation of the makespan.

**Fig. 5:**
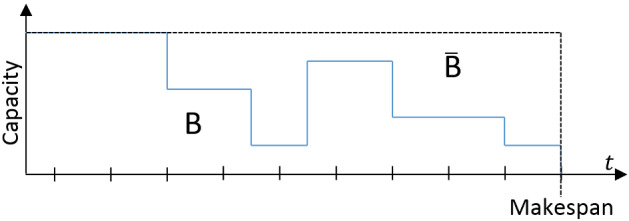
Visualization of the *B* and ${\bar{B}}$

Next, let us try to visualize with Fig. 5 the schedule of a DAG on an unrelated multiprocessor platform as a two-dimensional area. Let the *Capacity* be the sum of the speeds based on the task-processor mapping of the tasks that are in execution. The vertical axis shows the capacity. The horizontal axis is the time duration the application takes to complete its execution. Let *B* be the area where processors are busy executing some workload, and ${\bar{B}}$ be the area that the processors are idle. So the makespan is given by:

8 $$\begin{aligned} Makespan= \frac{B + {\bar{B}}}{Capacity} \end{aligned}$$

To compute the makespan for unrelated multiprocessors, we need to find the terms of Eq. (8) that maximize the numerator and minimize the denominator.

First, regardless of the execution time of the application, the total workload (${\mathcal {W}}_1$) is equal to the area in *B*. At run-time, a node can run with a speed that is lower than one (smaller vertical-value), which would have a proportionately longer duration (larger horizontal value), as we explained in Fig. 4. So, the area of *B*, which is composed of the workload of all the tasks, remains constant and is equal to the total workload of the DAG.

We can have different capacities during different stable time intervals because the speeds depend on the task-processor mapping. The largest possible capacity of the platform is $M$ when all the processors execute tasks with speed one. The smallest value is one, which is the case when one task is executing, and because the scheduler has the property of Lemma 1, it will schedule or migrate a task to its fastest (speed equal to one) available processor. To determine the capacity that would lead to the estimation of the makespan, we define as  the minimum capacity among all the task-processor mappings that we can have based on scheduling decisions of $\mathcal {GHE}$ for $m \le M$ processors.

To determine ${\bar{B}}$, we need the duration (horizontal value) that there are idle processors and the capacity of the unused processors (vertical value). Because we assume a work-conserving scheduler, we know that if there are idle processors and the application is not finished, there are still tasks that we need to execute, but they are restricted by their dependencies. Let  denote the workload of an arbitrary path of the application. The workload of any path in the DAG can be at most the workload of the critical path. The critical path length is different depending on the task-processor mapping of the tasks that belong to the critical path. The shortest length of the critical path is ${\mathcal {W}}_{\infty }$, which is the case if all task-processor mappings have speed one. Let $\tilde{\mathcal {O}^{}_{}}$ denote the speed that leads to the worst-case (i.e., maximum) unused capacity for all the tasks. Thus, the $\frac{{\mathcal {W}}_{\infty }}{\tilde{\mathcal {O}^{}_{}}~}$ shows the length of the critical path. Let ${\tilde{idle}}$ denote the unused capacity, and by replacing the terms to Eq. (8), we get:It can be seen from Eq. (7) that the values of $\tilde{\mathcal {O}^{}_{}}$ and ${\tilde{idle}}$ for a homogeneous multiprocessor platform are one and $(M-1)$, respectively. However, the values of $\tilde{\mathcal {O}^{}_{}}$ and ${\tilde{idle}}$ are unknown for unrelated multiprocessors. Because the scheduler is work-conserving ${\tilde{idle}}$ depends on $\tilde{\mathcal {O}^{}_{}}$. We combine these two parameters, and we call this expression heterogeneity. To find the maximum heterogeneity, we need to search among the different task-processor mappings that are determined by the $\mathcal {GHE}$ scheduler. To enumerate all possible task-processor mappings, we introduce:

#### Definition 6

Let $\pi _{}^{}$ be one permutation of *p* tasks selected from $N$ tasks of the application. The set of all the permutations of size *p* selected from $N$ different tasks is denoted by $\sigma _{p}$. The total number of permutations of size *p* selected from *N* tasks is $\frac{N!}{(N- p)!}$.

In Sect. 4.2, we define two ways to calculate the minimum capacity that takes into account the processor preference order of every task determined by the scheduler. In Sect. 4.3, we define heterogeneity among all the tasks and all the processors throughout the execution.

### Minimum capacity

For any stable time interval (based on Lemma 1), if $(x-1)$ processors are busy, then a task is executed at least on its $x^{th}$ faster processor. Let $\mathcal {O}^{\pi _{}^{}(k)}_{k}$ denote the $k^{th}$ speed of the $k^{th}$ task in permutation $\pi _{}^{}$. During a stable time interval when *x* processors are busy, we define in Eq. (9) the minimum capacity of the platform that tasks in permutation $\pi _{}^{} \in \sigma _{x}$ can execute with:

#### Definition 7

Capacity of *x* processors for permutation $\pi _{}^{}$:

9 $$\begin{aligned} S_{x}^{\pi _{}^{}} := \sum _{k=1}^{x} \mathcal {O}^{\pi _{}^{}(k)}_{k} \end{aligned}$$

The minimum capacity over all possible permutations $\pi _{}^{} \in \sigma _{M}$, denoted by $S_{M}^{}$, is given by:

#### Definition 8

Minimum capacity of the platform among all permutations:

10 $$\begin{aligned} S_{M}^{} := \min _{\pi _{}^{} \in \sigma _{M}} \{S_{M}^{\pi _{}^{}}\} \end{aligned}$$

The time complexity to evaluate Eq. (10) is exponential because we need to search through all the permutations. To avoid the high complexity, we trade off precision, and we define in Eq. (11) an alternative approach to calculating the minimum capacity for $1 \le m \le M$ processors. Instead of searching among all the permutations, we search among all the tasks to find the minimum capacity. This approach is always more or equally pessimistic compared to Eq. (10), and the proof is presented in Appendix A.1.

#### Definition 9

Minimum capacity of the platform among all tasks and processors:

11 $$\begin{aligned} S_{m}^{'} := \sum _{x=1}^{m} \min _{i=1}^{N} \{ \mathcal {O}^{i}_{x} \} \end{aligned}$$

While the capacity of the hardware platform for homogeneous and related machine models does not change from one task to another, the capacity for unrelated machines changes from one DAG to another, the definitions of capacity in Eqs. (10) and (11) are measures of the unrelated platform concerning the tasks, and they will be used in deriving the makespan of a DAG. Intuitively, the capacity shows the minimum rate that the workload of a DAG is executed for a given number of processors of the unrelated platform.

### Heterogeneity

In this subsection, we use the notion of heterogeneity to capture how much capacity of the platform could be wasted (recall the area of unused capacity), which will be used to find the makespan. For a permutation $\pi _{}^{}$, based on Lemma 1, if ($x-1$) processors are busy, a task in the worst-case will execute with speed $\mathcal {O}^{\pi _{}^{}(x)}_{x}$. Based on the $\mathcal {GHE}$, the unused capacity is given by $S_{M}^{\pi _{}^{}}$ -$S_{x}^{\pi _{}^{}}$ that depends on the speed $\mathcal {O}^{\pi _{}^{}(x)}_{x}$. To find the maximum unused-capacity area, for a permutation $\pi _{}^{}$ we define with Eq. (12) the heterogeneity. It finds for a specific permutation the maximum heterogeneity among the different processors.

#### Definition 10

Heterogeneity for permutation $\pi _{}^{}$:

12 $$\begin{aligned} \lambda _{}^{\pi _{}^{}} := \max _{x=1}^{M} \left\{ \frac{S_{M}^{\pi _{}^{}} -S_{x}^{\pi _{}^{}}}{\mathcal {O}^{\pi _{}^{}(x)}_{x}} \right\} \text { where, } \mathcal {O}^{\pi _{}^{}(x)}_{x} \ne 0 \end{aligned}$$

To identify the maximum permutation-based heterogeneity ($\lambda _{}^{}$) we define Eq. (13), where we search among all the permutations.

#### Definition 11

Maximum heterogeneity among all permutations:

13 $$\begin{aligned} \lambda _{}^{} := \max _{\pi _{}^{} \in \sigma _{M}} \{\lambda _{}^{\pi _{}^{}}\} \end{aligned}$$

Similarly, with the permutation-based capacity, from Eq. (10), the calculation of the maximum heterogeneity, from Eq. (13), has exponential time complexity because we need to search through all of the permutations. To avoid the high complexity, we define heterogeneity by searching through all of the tasks and all the processors instead of searching among all the permutations. If the workload on the critical path (${\mathcal {W}}_{\infty }$) is executed with speed $\mathcal {O}^{i}_{x}$ then the unused capacity is given by Eq. (14), where $\overline{\mathcal {O}}_{x}^{i}$ is the subset of $\mathcal {O}^{i}_{}$, whose speed is lower than the $x^{th}$ fastest processors for task $\tau ^{i}$.

#### Definition 12

Unused capacity if $\tau ^{i}$ is scheduled on processor *x*:

14 $$\begin{aligned} idle^{i}_{x} := \sum _{y \in \overline{\mathcal {O}}_{x}^{i}} \max _{j=1}^{N} \{ \mathcal {O}^{j}_{y} \} \end{aligned}$$

To find the heterogeneity ($\lambda _{}^{'}$), we combine Eq. (14) with its corresponding busy speed $\mathcal {O}^{i}_{x}$, and we maximize among the different tasks and different processors. The second version of heterogeneity is always more or equally pessimistic compared to Eq. (15), and we present the proof in Appendix (A.2).

#### Definition 13

Maximum heterogeneity among all tasks and processors:

15 $$\begin{aligned} \lambda _{}^{'} := \max _{i=1}^{N} \left\{ \max _{x=1}^{M} \left\{ \frac{idle^{i}_{x}}{ \mathcal {O}^{i}_{x}} \right\} \right\} \text { where, } \mathcal {O}^{i}_{x} \ne 0 \end{aligned}$$

For unrelated multiprocessors, it is not clear which path of the DAG would lead to the worst-case schedule length. However, in both approaches, to identify the heterogeneity, **all** the tasks and not just the tasks that belong to the critical path are considered. So the main idea is to combine heterogeneity, which takes into account all the possible tasks-processor mappings, with the critical path that has the largest workload among all the paths (Eq. (5)) to identify the maximum unused capacity area. Intuitively, the heterogeneity shows if the unrelated platform is appropriate for a DAG concerning an "ideal platform" with the same number of processors that all tasks will execute for their minimum WCET among the heterogeneous processors.

Next, we use the example of Fig. 2 to calculate the two versions of the capacity and the heterogeneity of the platform. First, we present the permutation-based approach. Each permutation is composed by two tasks that are executing to two processors, and let $\langle A,B \rangle$ denotes the permutation with tasks $\tau ^{A}$ and $\tau ^{B}$ that execute on two processors. For the application and the platform presented in Fig. 2, the total number of permutations is $N!/(N-M)! =6!/4! =30$. We present the calculation for permutations: $\langle A,B \rangle$, $\langle A,C \rangle$, $\langle A,D \rangle$, $\langle A,E \rangle$, and $\langle D,C \rangle$ and we omit the calculations of the remaining permutations. We select these permutations because there are representative since the tasks have similar WCET. For example, the calculations of the permutations $\langle A,E \rangle$ and $\langle A,F \rangle$ are the same because $\tau ^{E}$ and $\tau ^{F}$ have the same WCETs.

**Fig. 6:**
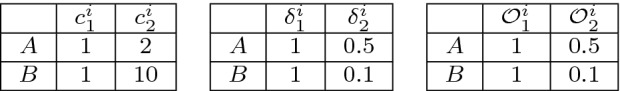
Permutation $$\langle A,B \rangle $$.  and

**Fig. 7:**
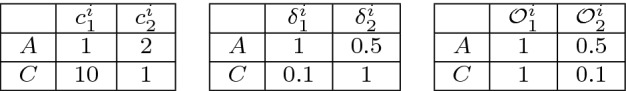
Permutation $$\langle A,C \rangle $$.  and

**Fig. 8:**
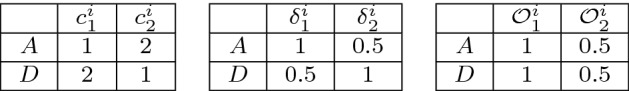
Permutation $$\langle A,D \rangle $$.  and

**Fig. 9:**
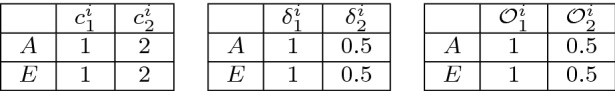
Permutation $$\langle A,E \rangle $$.  and

**Fig. 10:**
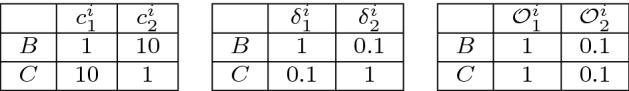
Permutation $$\langle B,C \rangle $$.  and

First, for each permutation, we present the table with the WCET of the tasks ($c^{i}_{x}$) that we consider for the specific permutation, presented at the left-hand side table at Figs. 6, 7, 8, 9 and 10. Next, at the tables which are at the center of Figs. 6–10 we compute the speed of the tasks ($\delta ^{i}_{x}$) for all the processors, based on Eq. (6). Next, based on the speeds of the tasks for the processors, at the right-hand side table of Figs. 6–10 we calculate the speed-preferences ($\mathcal {O}^{i}_{x}$) of the tasks that belong to the permutation for each processor based on Definition 5.

For every permutation, we calculate the permutation-based capacity ($S_{M}^{}$) and the heterogeneity ($\lambda _{}^{}$) based on Eqs. (9) and (12), respectively. The calculations to compute the capacity and the heterogeneity are presented at the captions of Figs. 6–10. By investigating all the permutation including the ones that we omitted for brevity, the minimum permutation-based capacity given by Eq. (10) is 1.1 and the maximum permutation-based heterogeneity given by Eq. (13) is 0.5.

**Fig. 11:**
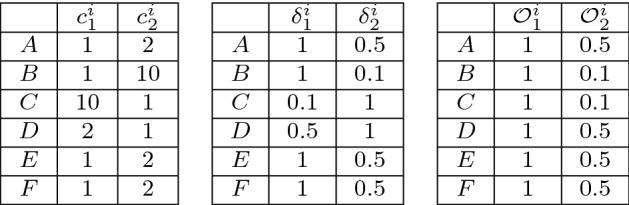
The three tables are WCETs (left-hand side), speeds (center), and preference of speeds (right-hand-side) of all the application tasks that we need for the fast approach

To avoid the high time complexity of enumerating all the permutations, we apply Eqs. (11) and (15) to find the capacity ($S_{M}^{'}$) and the heterogeneity ($\lambda _{}^{'}$) in polynomial time complexity. At Fig. 11, we present the table with the WCET ($c^{i}_{x}$), the speed ($\delta ^{i}_{x}$) and the preference of speeds ($\mathcal {O}^{i}_{x}$) for all the tasks of application that is presented in Fig. 2.

To find the capacity of the two processors first, we compute the minimum speeds that the tasks have based on the greediness property for the two processors that can execute. We compute the minimum speed of the first speed preference $\min _{i=1}^{N} \{\mathcal {O}^{i}_{1}\} =1$, and the second speed preference $\min _{i=1}^{N} \{\mathcal {O}^{i}_{2} \}= 0.1$ among all the tasks and the capacity is given by: $S_{M}^{'} = 1+0.1 = 1.1$. To find the heterogeneity (recall that the scheduler is work-conserving), we need to check only the case that one out of the two processors is idle. Also recall that the scheduler has the greediness property, and therefore, we need to find the maximum speed that remains idle among the processors that are in the list of second-speed preference of the task (last column, right-hand side table in Fig. 11). We compute the maximum speed that is not used in case one processor is idle as follows $\max _{i=1}^{N} \{\mathcal {O}^{i}_{2} \}= 0.5$ and we get $idle^{max}_{2} = 0.5$. Since the speed of the first speed preference of all the tasks is one, the heterogeneity is $\lambda _{}^{'} = \frac{0.5}{1} = 0.5$.

## Makespan calculation

This section presents two approaches to calculate the makespan of a DAG on unrelated multiprocessor platforms. Initially, Sect. 5.1 provides the proof sketch of the proposed approaches. The first issue (Sect. 5.2) for both approaches is how to determine a formally proven upper bound on the execution time of the DAG (a requirement on safety). A makespan computation must never underestimate the length of the schedule to ensure that the real-time constraints are satisfied. Lemma 2 presents an exhaustive search-based combinatorial approach to calculate such an upper bound on the makespan. A safe upper bound on the makespan is determined by identifying all the permutations of possible task-processor mappings. This approach has exponential time complexity but provides a tight makespan estimation (as will be evident later in our experiments). As a result, such an approach can be used only for a small number of processors and tasks.

The second issue (Sect. 5.3) is to provide a tight estimation of the makespan that avoids exhaustive approaches and can be calculated efficiently. Theorem 1 proposes a polynomial time complexity makespan calculation. Based on Lemmas 3 and 4, it is proven that the makespan calculated by Theorem 1 is always greater or equal than the (exhaustive) makespan given by Lemma 2 but still quite tight. As a result, the second approach is shown to be always a safe estimation of the makespan.

### Overview

To find a safe and tight bound on the makespan, we partition an arbitrary schedule across different time intervals such that the number of busy processors in each such interval is constant. We use an exhaustive approach, and for every interval, we search through all of the permutations of task-processor mappings. To identify for every interval the permutation that leads to the worst-case schedule length, we adapt for the unrelated model (minimum capacity and heterogeneity) two well-known parameters used earlier in the context of related multiprocessors (Funk et al. 2001; Jiang et al. 2017). These two parameters that try to maximize the schedule length are combined with the total workload and the workload of the critical path of the DAG to compute the makespan.

The initial, exhaustive approach has exponential time complexity since all the permutations need to be searched. As a result, it is applicable to a restricted number of tasks and processors. To address this limitation, we propose a polynomial time-complexity version of the heterogeneity and capacity that is independent of the permutations and formally proven to be always more pessimistic compared to the permutation-based version of the parameters.

### Exhaustive search makespan

In this section, we present our first result in Lemma 2 that can be used to compute the makespan. The final formula of the makespan uses the total workload and the workload of the longest path presented in Sect. 2 and the permutation-based minimum capacity and heterogeneity introduced in Sects. 4.2 and 4.3, respectively.

#### Lemma 2

Exhaustive makespan ( Comb ): The makespan of a DAG executed on an unrelated multiprocessor platform is given by:16

#### Proof

Let $B_p$ denote the sum of the lengths of the time intervals where exactly *p* processors are busy. Because $\mathcal {GHE}$ is work-conserving, we know that there will always be at least one processor busy during the execution of the application. So for the makespan , we have:17For an application that is executed on an unrelated multiprocessor platform, the different task-to-processor mappings can lead to different schedule lengths. To describe all the task-processor mappings when exactly *p* processors are busy, we introduce $B_p^{\pi _{}^{}}$, which denotes the time interval when exactly *p* processors are busy by the tasks of permutation $\pi _{}^{}$ of size *p*. Since we can have different permutations that occupy *p* processors, it holds that:

18 $$\begin{aligned} B_p = \sum _{\pi _{}^{} \in \sigma _{p}} B_p^{\pi _{}^{}} \end{aligned}$$

where $\sigma _{p}$ is the set of all permutations of size *p*. If during run-time a permutation $\pi _{}^{\prime }$ does not appear in the schedule, then it holds that $B_p^{\pi _{}^{\prime }}=0$.

Consider an arbitrary schedule and an arbitrary busy interval $B_p^{\pi _{}^{}}$. Based on Lemma 1, during $B_p^{\pi _{}^{}}$, the $p^{th}$ task belonging to $\pi _{}^{}$ in the worst-case will be executed with speed $\mathcal {O}^{\pi _{}^{}(p)}_{p}$ where $\mathcal {O}^{\pi _{}^{}(p)}_{p} \ne 0$. Let  denote the total amount of workload completed at speed $\mathcal {O}^{\pi _{}^{}(p)}_{p}$ from task $\tau ^{\pi _{}^{}(p)}$ that belongs to an arbitrary path $\gamma$ and at the $p^{th}$ position of permutation $\pi _{}^{}$ and we have:We break up the workload of the tasks that belong to the critical path ${\mathcal {W}}_{\infty }$ into fragments that depend on the task-processor mappings; that is, fragments that depend on permutations $\pi _{}^{}$ of size *p* denoted by ${\mathcal {W}}_{\infty }^{p,\pi _{}^{}}$. If for a permutation $\pi _{}^{}$, it holds that $B_p^{\pi _{}^{}}=0$, then it also holds that ${\mathcal {W}}_{\infty }^{p,\pi _{}^{}}=0$ because this permutation did not appear in the schedule, so it does not have any workload. With ${\mathcal {W}}_{\infty }^{p}$, we denote the workload of the critical path of all permutations executed by the same number of processors. To collect the workload from all the permutations, we define:

19 $$\begin{aligned} {\mathcal {W}}_{\infty }\ge \sum _{p=1}^{M} {\mathcal {W}}_{\infty }^{p} = \sum _{p=1}^{M} \sum _{\begin{array}{c} \pi _{}^{} \in \sigma _{p} \wedge \\ B_p^{\pi _{}^{}} \ne 0 \end{array}} {\mathcal {W}}_{\infty }^{p,\pi _{}^{}} \end{aligned}$$

Since the critical path is $cp$ with total workload ${\mathcal {W}}_{\infty }^{p,\pi _{}^{}}$ belonging to permutation $\pi _{}^{}$, the actual workload is bounded as follows:  and we have:

$$\begin{aligned} B_p^{\pi _{}^{}} \cdot \mathcal {O}^{\pi _{}^{}(p)}_{p} \le {\mathcal {W}}_{\infty }^{p,\pi _{}^{}} \end{aligned}$$

If there is at least one processor idle ($p<M$) and there are still tasks that we need to execute, then they must be restricted by their dependencies. On unrelated multiprocessors, due to the different task-processor mappings, the execution time of any path can vary. So it is not clear which path is going to determine the makespan of the DAG. To identify the worst-case scenario to find a safe upper bound on the makespan, we consider two pessimistic but safe characteristics of the DAG. First, we consider the path with the largest workload that will guide the length of the schedule. Second, with the use of heterogeneity, we consider the worst-case mapping among all the tasks that would lead to the largest unused capacity throughout the execution. The largest unused capacity throughout the execution depends on two factors: 1) the unused processors and 2) the duration that these processors are idle. More precisely, to find the duration of the critical path workload, we assume, based on Lemma 1, that a task that belongs to the critical path is executing at speed $\mathcal {O}^{\pi _{}^{}(p)}_{p}$. If $\mathcal {O}^{\pi _{}^{}(p)}_{p}$ is busy then the unused capacity is given by $S_{M}^{\pi _{}^{}}-S_{p}^{\pi _{}^{}}$. With the heterogeneity given by Eq. (12), we can find the worst-case for a specific permutation because it maximizes these two factors among all the task-processor mappings. By replacing the $\mathcal {O}^{\pi _{}^{}(p)}_{p}$ we have:

20 $$\begin{aligned} \implies B_p^{\pi _{}^{}} \cdot \frac{S_{M}^{\pi _{}^{}}-S_{p}^{\pi _{}^{}}}{\lambda _{}^{\pi _{}^{}}} \le {\mathcal {W}}_{\infty }^{p,\pi _{}^{}} \end{aligned}$$

By Eq. (19) and since for an arbitrary $\pi _{}^{}$ it holds that $\lambda _{}^{\pi _{}^{}} \le \lambda _{}^{}$ we have:

$$\begin{aligned}&\sum _{p=1}^{M}\sum _{\pi _{}^{} \in \sigma _{p} } B_p^{\pi _{}^{}} \cdot \frac{S_{M}^{\pi _{}^{}} - S_{p}^{\pi _{}^{}}}{\lambda _{}^{}} \le \sum _{p=1}^{M} \sum _{\begin{array}{c} \pi _{}^{} \in \sigma _{p} \wedge \\ B_p^{\pi _{}^{}} \ne 0 \end{array}} {\mathcal {W}}_{\infty }^{p,\pi _{}^{}}\\&\sum _{p=1}^{M-1}\sum _{\pi _{}^{} \in \sigma _{p} } B_p^{\pi _{}^{}} \cdot \frac{S_{M}^{\pi _{}^{}} - S_{p}^{\pi _{}^{}}}{\lambda _{}^{}} \le {\mathcal {W}}_{\infty }\end{aligned}$$

Equivalently,

21 $$\begin{aligned} \sum _{p=1}^{M-1}\sum _{\pi _{}^{} \in \sigma _{p} } B_p^{\pi _{}^{}} \cdot (S_{M}^{\pi _{}^{}} - S_{p}^{\pi _{}^{}}) \le \lambda _{}^{} \cdot {\mathcal {W}}_{\infty }\end{aligned}$$

During $B_p^{\pi _{}^{}}$ the *p* processors are busy with platform capacity $S_{p}^{\pi _{}^{}}$, which means that after $B_p^{\pi _{}^{}}$ time units, the amount of workload that is done is $B_p^{\pi _{}^{}}\cdot S_{p}^{\pi _{}^{}}$. Since the application completes when no processor is busy, the total workload is given by ${\mathcal {W}}_1= \sum _{p=1}^{M} \sum _{\pi _{}^{} \in \sigma _{p}} B_p^{\pi _{}^{}} \cdot S_{p}^{\pi _{}^{}}$. By adding this term in both sides in Eq. (21), we get:

$$\begin{aligned}&\sum _{p=1}^{M-1}\sum _{\pi _{}^{} \in \sigma _{p} } [ B_p^{\pi _{}^{}} \cdot (S_{M}^{\pi _{}^{}} - S_{p}^{\pi _{}^{}}) + B_p^{\pi _{}^{}} \cdot S_{p}^{\pi _{}^{}} ] + \sum _{\pi _{}^{} \in \sigma _{p}} B_p^{\pi _{}^{}} \cdot S_{M}^{\pi _{}^{}} \le {\mathcal {W}}_1+ \lambda _{}^{} \cdot {\mathcal {W}}_{\infty }\\&\sum _{p=1}^{M-1}\sum _{\pi _{}^{} \in \sigma _{p} } B_p^{\pi _{}^{}} \cdot S_{M}^{\pi _{}^{}} + \sum _{\pi _{}^{} \in \sigma _{p}} B_p^{\pi _{}^{}} \cdot S_{M}^{\pi _{}^{}} \le {\mathcal {W}}_1+ \lambda _{}^{} \cdot {\mathcal {W}}_{\infty }\\&\sum _{p=1}^{M}\sum _{\pi _{}^{} \in \sigma _{p} } B_p^{\pi _{}^{}} \cdot S_{M~}^{\pi _{}^{}} \le {\mathcal {W}}_1+ \lambda _{}^{} \cdot {\mathcal {W}}_{\infty }\end{aligned}$$

Based on the definition of $S_{M}^{}$, given by Eq. (10) we have $S_{M~}^{\pi _{}^{}} \ge S_{M}^{}$ and by the definition of $B_p$ given by Eq. (18), we have:

$$\begin{aligned} \implies&\sum _{p=1}^{M} B_p \cdot S_{M}^{} \le {\mathcal {W}}_1+ \lambda _{}^{} \cdot {\mathcal {W}}_{\infty }\\&\sum _{p=1}^{M} B_p \le \frac{{\mathcal {W}}_1+ \lambda _{}^{} \cdot {\mathcal {W}}_{\infty }}{S_{M}^{}} \end{aligned}$$

Since the scheduler is work-conserving it holds that  and consequently:This completes the proof of Lemma 2. $\square$

For the example that is given in Fig. 2 we replace the parameters to the equation given in Lemma 2 and we get the makespan .

### Efficient makespan

The calculation of the capacity and heterogeneity of the platform using the permutation-based parameters has exponential time complexity. With Lemmas (3) and (4), we prove that the permutation-independent parameters always provide more pessimistic minimum capacity and heterogeneity. The proofs are given in Appendix A. The final formula of the efficient makespan uses the total workload and the workload of the longest path presented in Sect. 2 and the minimum capacity and heterogeneity that are independent of the permutations that were presented in Sects. 4.2 and 4.3, respectively.

#### Lemma 3

The $S_{M}^{'}$ is always less or equal to the minimum capacity $S_{M}^{}$ between the different permutations.

22 $$\begin{aligned} S_{M}^{} \ge S_{M}^{'} \end{aligned}$$

#### Lemma 4

The $\lambda _{}^{'}$ is always greater or equal to the maximum heterogeneity $\lambda _{}^{}$ between the different permutations.

23 $$\begin{aligned} \lambda _{}^{} \le \lambda _{}^{'} \end{aligned}$$

#### Theorem 1

Efficient makespan ( Fast ): The makespan of a DAG executed on an unrelated multiprocessor platform is given by.24

#### Proof

From Lemmas 3 and 4 it follows that the makespan calculated by using $\lambda _{}^{'}$ and $S_{M}^{'}$ is always larger in comparison to the makespan calculated from Lemma 2. Since the makespan  from Lemma (2) is safe, the makespan  given by Eq. (24) is also safe (i.e., an upper bound). $\square$

We compare the upper bound on the makespan that we computed to the optimal schedule length, i.e., minimum completion time, denoted by  to theoretically evaluate how much pessimism is introduced to our makespan computation compared to . Finding the  is intractable (Garey and Johnson 2002), and we compute a lower bound on the  as follows. First, we optimistically assume that the $\mathcal {GHE}$ finds a processor that provides speed one for every task of the DAG. Thus, each task is executed for its minimum WCET. Let an upgraded DAG, denoted by ${\hat{G}}$, be an isomorphic DAG to $G$, meaning that $V{} = {\hat{V}}$ and $E= {\hat{E}}$, where for every task instead of having $M$ WCETs, each task has only one WCET, the $c^{i}_{min}$. Because its task has one WCET, the homogeneous setup Graham (1969); Brent (1974); Blumofe and Leiserson (1999) can be applied. A lower bound on the optimal schedule length is computed by $LB = \max \{{\hat{{\mathcal {W}}_{\infty }}}, \frac{{\hat{{\mathcal {W}}_1}}}{M}\}$ for the upgraded DAG ${\hat{G}}$, which is also a lower bound for the original DAG $G$.

#### Corollary 1

The makespan given by Theorem 1 is $(\frac{ M+ \lambda _{}^{'}}{S_{M}^{'}})$ times larger than the optimal schedule length ():

#### Proof

From Eq. (24), given by Theorem 1, we have:By definition , so it holds that  and .25$\square$

The value of $(\frac{ M+ \lambda _{}^{'}}{S_{M}^{'}})$ depends on the WCETs of the tasks for the processors of the platform. If all the speeds for all the tasks are one, the platform specialize to homogeneous multiprocessors (Eq. (7)) (Graham 1969; Brent 1974; Blumofe and Leiserson 1999) and the value (a.k.a approximation, speed-up, and resource augmentation factor) is $(2 - \frac{1}{M})$. In conclusion, the upper bound on the makespan computed by Eq. (24) is $(\frac{ M+ \lambda _{}^{'}}{S_{M}^{'}})$ times larger compared to the optimal schedule length of any scheduling heuristic that has the work-conserving property and the greediness property.

### Summary

This section presents two approaches to calculate the makespan of a DAG on unrelated heterogeneous multiprocessors. Initially, we present an exhaustive permutation-based approach (Comb) that safely calculates the makespan and has exponential time complexity (Lemma 2). Then we use Comb as the stepping stone to develop Fast that is given in Theorem (1) to find the makespan in polynomial time. The main advantage of the approach is its generality. The assumptions regarding the platform model can cover a wide range of heterogeneous multiprocessors. The DAG model can be applied to a broad range of parallel applications, such as OpenMP task-based parallel applications. Finally, keeping the scheduler general, assuming only that it is work-conserving also allows us to cover a broad class of schedulers and not just one scheduling policy.

## Complexity analysis

Initially, we analyze the parameters that are common for all the proposed bounds. Next, we show that the Comb has exponential time complexity and Fast has polynomial time complexity.

Common: For a specific task, the minimum WCET between the processors can be calculated in $O(M)$. The critical path can be calculated in $O(|V|+|E|)$ with the use of topological sort (West et al. 2001). As a result, ${\mathcal {W}}_{\infty }$ can be calculated with $O(\texttt {max}\{(|V|+|E|), (M))\}$ time complexity. The total work is the sum of the minimum WCET of all tasks, so ${\mathcal {W}}_1$ is computed with $O(\texttt {max}\{N,M\}$) time complexity.

Comb : The parameter $\sigma _{p}$ of $\lambda _{}^{}$ and the parameter $S_{M}^{}$ are calculated by enumerating all the permutations of the tasks ($N$) to processors ($M$). Thus, $\sigma _{p}$ is of size $O(N^{M})$ and as a result Comb, given by Lemma 2, has a time complexity of $O(N^{M})$. So, the exhaustive approach has exponential time complexity.

Fast: The makespan  is given by Theorem 1 and it uses the parameters $S_{M}^{'}$ and $\lambda _{}^{'}$ that are independent of the permutations and can be calculated in polynomial time. Initially, $\mathcal {O}^{i}_{}$ requires time $O(M\cdot log(M))$ to sort the array of speeds. We have to calculate $\mathcal {O}^{i}_{}$ for all the tasks, so the time complexity is $O(N\cdot M\cdot log(M))$ using an efficient sorting algorithm like Heapsort. However, accessing $\mathcal {O}^{i}_{x}$ and $\overline{\mathcal {O}}_{x}^{i}$ requires constant time because the array is sorted. Next, the complexity of computing $S_{M}^{'}$ is $O(\texttt {max}\{N, M\})$ because to identify the minimum requires $O(N)$ and the sum includes all the processors ($M$). Furthermore, the calculation of the heterogeneity requires the parameter $idle^{i}_{x}$ that has time complexity $O(\texttt {max}\{N\cdot M\})$, because the maximum requires $O(N)$ and the sum is over at most $M-1$ iterations. Finally, the heterogeneity $\lambda _{}^{'}$ uses $idle^{i}_{x}$ together with two maximum operations, that can be calculated in $O(N)$ and $O(M)$, respectively. So, $\lambda _{}^{'}$ is calculated with time complexity $O(\texttt {max}\{N^{2}, M^{2}\})$, which is polynomial. As a result, for the Fast, we have $O( \texttt {max}\{(|V|+|E|), (M)\} + \texttt {max}\{N,M\} + \texttt {max}\{N, M\} + N\cdot M\cdot log(M) + \texttt {max}\{N^{2}, M^{2}\})$, that is, $O(\texttt {max}\{N^{2}, M^{2}\})$, which is polynomial in time complexity.

## Evaluation

To quantitatively evaluate the proposed makespan calculation, Sect. 7.1 presents the simulation framework, and Sect. 7.2 presents the simulation results for different parameters of our model for four OpenMP parallel applications and synthetic DAGs.

### Simulation framework

First, we present the method by which we model the DAGs of the applications and the synthetic workloads. Next, we describe the simulator that is used to calculate the makespan. Finally, we describe the configuration of the applications and the evaluation metrics used.

#### DAG modeling

The WCET of a task is generated by adding a randomly generated value to the $c^{i}_{min}$ (minimum WCET between the different processor types). With the parameter $Limit$, we limit the range of the randomly generated values. More formally, the WCET of every task is given by, $c^{i}_{t} = c^{i}_{min} + Rand(0, Limit)$. The exact value of $c^{i}_{min}$ is stated in the experimental section. We perform two types of experiments where we consider real applications and synthetic DAGs.

**Applications** We model the DAG of four parallel, task-based OpenMP applications from the BOTS benchmark suite (Duran et al. 2002): Fibonacci, Sort, Strassen, and FFT. Fibonacci has a tree-like structure and is a good representative of many recursive applications. It is simple and is very helpful for the understanding of the parallel execution of the tasks. Sort is a common operation in almost all fields of computing. Strassen is an efficient matrix-multiplication algorithm that is used in many scientific applications. Finally, FFT is used in signal and image processing. The analysis of the OpenMP code for each application is performed manually. Then the applications are implemented in our simulation framework to generate the DAG automatically. Initially, we categorize the parts of the code based on their functionality, and we introduce three nodes:

- **Spawn nodes** The keyword *#omp pragma task* of a loop generates multiple tasks. The spawn node models the cost of parallel work generation.
- **Basic nodes** It models the execution time of a sequentially executed code, which is the actual work of the parallel application.
- **Synchronization nodes** We use synchronization nodes to model the *#omp pragma taskwait*. A Synchronization node models the cost (in time) of the synchronization.

For Fibonacci, Strassen, and FFT, the structure of the DAG depends on the input size. The structure of the DAG for Fibonacci depends on the actual value, and for Strassen and FFT, it depends on the array size. For Sort, the DAG structure is data-dependent; for the same array size but different actual data, we can have different DAGs. Previous work introduced conditional nodes to express the alternative execution paths. We use the method in Baruah et al. (2015b) to transform the conditional DAG to a non-conditional worst-case DAG for Sort. An example of DAG modeling can be found in Voudouris et al. (2017).

The applications under analysis have thousands of tasks. However, we note that the applications have only a few different tasks that perform the same function, and, as a result, they have the same set of WCETs. Tasks with the same WCET for the various processors will lead to the same permutations. Consequently, we need to calculate all the permutations only based on the unique tasks, which, in practice, has exponential time complexity to the number of unique tasks rather than the number of tasks. For example, Fibonacci has 32836 tasks for input 20, but there is only one unique task that calculates the Fibonacci numbers. Similarly, for Sort, FFT, Strassen, there are 2, 3, and 1 unique task, respectively. In Chronaki et al. (2015), they consider OmpSs applications, which are similar to the OpenMP applications. These applications have few unique tasks compared to the total number of tasks: Cholesky factorization, QR factorization, Heat diffusion, and Integral Histogram have 4, 4, 3, and 2 unique tasks, respectively, for DAGs with a few thousands of tasks. Although there are three categories of each node (spawn, base, and synchronization), the total number of possible pairs of tasks and node categories is significantly fewer than the total number of tasks.

**Synthetic DAGs** A synthetic DAG is modeled by following a similar structure of the applications. We generate a fully-balanced tree together with the mirror tree for the *Sync* nodes. The maximum degree of the *Spawn* nodes, and the maximum height of the DAG can be set as parameters. A time budget is assigned to every *Spawn* node, which is responsible for distributing it to its child nodes and the corresponding *Sync* node to get the desirable ${\mathcal {W}}_1$ and ${\mathcal {W}}_{\infty }$ characteristics of the DAG. Next, the number of task types is given as a parameter to the DAG. We randomly generate WCETs with the use of the $Limit$ parameter with the same approach that we use for the real applications.

#### Simulator

The simulator is event-based, where an event is considered the completion of the execution of the tasks, and we implement the scheduler described in Sect. 2. The real applications have many tasks, so to avoid state-space explosion, we generate the DAG gradually. We follow the schedule of the DAG, assuming that all tasks are executed for their WCET, and we monitor its execution for two independent schedules/runs. First, we schedule the DAG under consideration with infinite processors (in practice: $INT\_MAX$, in C++) to calculate the ${\mathcal {W}}_1$ and ${\mathcal {W}}_{\infty }$ parameters given in Sect. 2. Next, for the second schedule/run, we set the number of processor types and the total number of processors that we want to test. We make sure that there is at least one processor of each type, and we use random assignment of the processors to the processor types. We schedule the DAG, and we monitor the unique tasks to determine the capacity and the heterogeneity for the Fast given and for Comb makespan given in Sects.  4.2 and 4.3. Based on Lemma (2) and Theorem (1), we use the parameters provided by the simulator to calculate the makespan for  and . The schedule-length of the second run ($Sim$) is an instance of the DAG execution and cannot be used as a safe estimation of the makespan due to timing anomalies; however, it can be seen as a lower bound on the best achievable makespan.

#### Configuration and evaluation metrics

Table 3 presents the configuration of the applications. Initially, the $c^{i}_{min}$ of the *Spawn*, *Base* and *Sync* are set to 300, 400 and 100 time units. The columns are the applications (Fibonacci, Sort, Strassen, and FFT). The first row is the input of the applications and the second row is the total number of nodes that the applications have. The third row is the total work (${\mathcal {W}}_1$), and the fourth row is the workload of the critical path (${\mathcal {W}}_{\infty }$) of the applications. The fifth row shows the ratio of the workload of the critical path to the total workload, and the last row shows the number of unique tasks.

**Table 3:** Application configurations

	Fib	Sort	Strassen	FFT
Input	20	32,768	512	8192
$\#$Nodes	32,836	16,043	22,410	23,748
${\mathcal {W}}_1$	8,756,400	4,403,300	7,843,300	6,221,400
${\mathcal {W}}_{\infty }$	8000	14,900	2500	5,1020
$\frac{{\mathcal {W}}_{\infty }}{{\mathcal {W}}_1}$	0.0009	0.003	0.0003	0.008
$\#$Unique tasks	3	6	3	9

To the best of our knowledge, no other related work provides a closed-form solution for the makespan calculation and an exact makespan of parallel applications modeled as DAGs on an unrelated multiprocessor platform. For our simulations, we use the following evaluation metrics:

**Tightness** The tightness is defined as the ratio . The exhaustive makespan calculations  given by Lemma 2 is compared to the  makespan given by Theorem 1.

**Pessimism** We derive a lower bound on the makespan by simulating the parallel applications’ actual execution with the $\mathcal {GHE}$ scheduler, where all the tasks are executed for their WCET. Let $Sim$ be the schedule length of the execution. The pessimism of our approach is defined as the ratio of . Note that even the optimal way to find the makespan has a length not smaller than $Sim$.

All the experiments are performed 100 times, and we report the average.

### Quantitative results


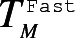
 is proven to be a safe makespan by showing that it is always greater than . As a result, our evaluation needs to quantify the overestimation introduced to avoid the exponential time complexity of the  approach. Consequently, the closer the estimation of  is to the estimation of , the better is the estimation. Next, by comparing our proposed approach with that of the simulation of the execution, we try to quantify the pessimism that is introduced compared to the best achievable makespan estimation. Ideally,  and  are equal and as close as possible to the lower bound of the best achievable makespan. By using the evaluation metrics defined in Sect. 7.1.3, we present the simulation results concerning different parameters of our model. Sections 7.2.1–7.2.3 show the results considering the DAG of the applications. Sections 7.2.4 and 7.2.5 present the result of synthetic DAGs.

**Fig. 12:**
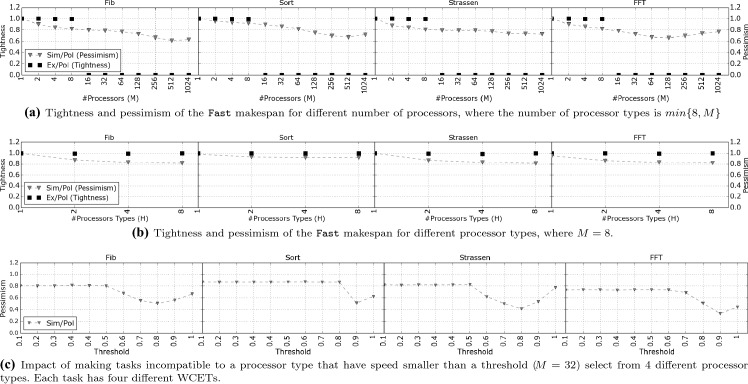
Simulation results of the applications

#### Impact of changing the number of processors

Figure 12a presents the tightness (Y-axis on the left-hand side) and the pessimism (Y-axis on the right-hand side) of Fibonacci, Sort, Strassen, and FFT as a function of the number of processors ($M$), where the number of processor types is up to $min\{8, M\}$. The points without a dashed line correspond to the tightness of the makespan. The points with a dashed line correspond to the pessimism. In this graph, the closer to one the values are, the better is the tightness, and the less is the pessimism.

The makespan calculation of  has polynomial time complexity, so we generate the results for up to a total of 1024 processors. On the contrary, the makespan calculation of  is the permutation-based approach which has exponential time complexity. With our simulation setup, we can simulate only up to 8 processors. The $Limit$ is set to 100 for this experiment.

Initially, it can be seen that for one processor, all the approaches are equal to ${\mathcal {W}}_1$. Furthermore, we can see that for up to eight processors, the tightness (the overestimation of the makespan) of  compared to  is less than $1\%$ on average and up to $1.2\%$ greater for all the applications. We have performed the same simulations, but with $Limit$ equal to 500 and 1000 (not shown in the plots). The average tightness of the makespan is slightly higher than $1\%$ and up to $3\%$. Next, it can be noted that by increasing the number of processors exponentially, the pessimism increases linearly. Compared to $Sim$, the pessimism we have averaged from $25\%$ up to $62\%$.

#### Impact of changing the number of processor types

Figure 12b presents the tightness and the pessimism for the different number of processor types for eight processors and the four applications. The horizontal axis is the number of processor types for the four applications, the left vertical axis is the tightness, and the right vertical axis is the pessimism. The $Limit$ for the random generation of the WCET is set to 100.

The tightness of , compared to the two permutation-based approaches, is, on average, $1\%$ and maximally $1.3\%$. Consequently, the margin between the polynomial and the exponential approach is not significant. Since we do not distinguish between the processor types for the calculation of heterogeneity and capacity in the polynomial approach but we consider the total number of processors, and it is expected to have similar behavior with the results shown in Fig. 12a. Next, we note that by increasing the number of processor types while the total number of processors remains the same, the pessimism increases since a relatively smaller number of tasks are now executing with a speed of one, which leads to a longer makespan.

Compared to $Sim$, we have, on average, $13\%$ and up to $23\%$ more pessimism. *Sim* is a lower bound on the optimal makespan, so if an exact makespan can be calculated for parallel applications, which is very unlikely to happen, our analysis can still provide an upper bound on makespan, which is at most $23\%$ longer than the optimal makespan. Therefore, we think our approach to finding the makespan using  is quite effective for applications that we have considered from the BOTS benchmark suite.

#### Impact of task-processor compatibility

Whether a task is compatible with a processor type or not is determined using a threshold speed for each experiment. If the initial speed of a task on a given processor is smaller than the threshold, its speed on that processor is set to zero ($\delta ^{i}_{t}=0$).

Figure 12c shows the compatibility of the tasks to the processors. The horizontal axis presents the speed threshold for Fibonacci, Sort, Strassen, and FFT. The vertical axis shows the pessimism of  with respect to $Sim$. The platform has 32 processors and four processor types. $Limit$ is set to 100 for this experiment.

Initially, for threshold 0.1, all the applications have pessimism only around $1/0.8=25\%$, i.e., the computed makespan is no more than 1.25 times greater than the optimal. In such a case, the scheduler can almost always find some compatible idle processor due to a relatively low threshold speed. Next, it can be seen that for all the applications, as the threshold increases, i.e., relatively more incompatible tasks, the pessimism initially remains constant and then increases since fewer compatible processors are available for the tasks to execute.

Next, we note that the pessimism starts to increase for Fibonacci and Strassen from speed threshold 0.5 while for Sort and FFT, the tightness begins to decrease after 0.8 and 0.7, respectively. Since Fibonacci and Strassen have fewer task types, 3 task types each, compared to Sort and FFT that have 6 and 9, respectively, more tasks are characterized as incompatible for Sort and FFT. As a result, more tasks have fewer processors to be executed for Sort and FFT. For Fibonacci and Strassen, tightness reaches its minimum value at 0.8 and for Sort and FFT at 0.9. There are many incompatible processors at that point, but because the platform has many processors, the scheduler can find available processors to schedule the tasks in parallel. However, we can see that the pessimism decreases for high thresholds since the scheduler (i.e., simulated schedule) cannot find available processors to schedule the tasks, and the total execution of the schedule increases. Consequently, the pessimism compared to $Sim$ decreases.

#### Impact of processor heterogeneity

**Fig. 13:**
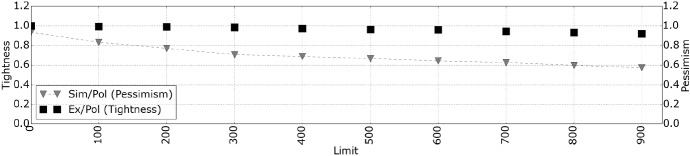
Tightness and pessimism for different variations of the WCET

To analyze in more detail the variation of the WCET of a task among the different processor types, we consider a synthetic DAG, and we vary the $Limit$ factor. Figure 13 presents the tightness and the pessimism for different values of the $Limit$. The horizontal axis is the $Limit$, the left vertical axis presents the tightness, and the right vertical axis is the pessimism. The platform has four processors and two processor types. The synthetic DAG has ${\mathcal {W}}_1=191400$ and ${\mathcal {W}}_{\infty }=5800$ for 938 nodes and 3 task types with ratio $\frac{{\mathcal {W}}_{\infty }}{{\mathcal {W}}_1}=0.03$. Note that this ratio is one to two orders of magnitude higher than the BOTS applications, so the impact of heterogeneity should be higher. Note that with $Limit=1000$, we can have a variation on the WCET from 2.5*x* to 10*x* for *Spawn* and *Sync* nodes, respectively, that have 400 and 100 time units for their $e_i^{min}$ values. We intentionally use extreme values to expose the limitations of .

By increasing $Limit$, which can be seen as making the platform more heterogeneous, the tightness of the  approach decreases. On average, we have $5\%$ and a maximum $11\%$ less tight makespan compared to exhaustive approaches. Such an increase in the makespan is due to the calculation of the heterogeneity $\lambda _{}^{'}$, which is calculated between all the tasks.  uses $\lambda _{}^{}$ which is calculated based on all of the permutations of tasks. We can see that the pessimism of  compared to $Sim$ increases as $Limit$ increases since more processors would have a lower speed. As a result, the makespan of  increases. Compared to $Sim$, we have on average $51\%$ and up to $74\%$ more pessimism. Note that although such values may be quite high for our analysis, we would like to stress that the degree of heterogeneity for higher $Limit$ is quite pessimistic for many practical heterogeneous platforms.

#### Impact of application characteristic

**Fig. 14:**
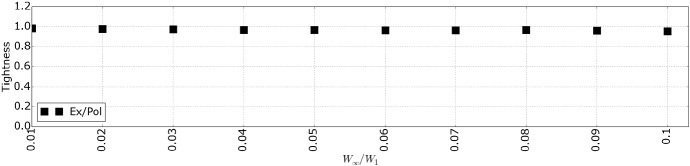
Comparison of the proposed methods for different characteristic of the synthetic DAG by varying the $\frac{{\mathcal {W}}_{\infty }}{{\mathcal {W}}_1}$ ratio

For this experiment, we characterize a DAG by ${\mathcal {W}}_1$ and ${\mathcal {W}}_{\infty }$ only, and we vary the characteristic (i.e., $\frac{{\mathcal {W}}_{\infty }}{{\mathcal {W}}_1}$) of an application. Note that $\frac{{\mathcal {W}}_{\infty }}{{\mathcal {W}}_1}$ is within (0, 1). If $\frac{{\mathcal {W}}_{\infty }}{{\mathcal {W}}_1} \approx 0$, then it means that the length of the critical path is much smaller in comparison to that of the total work (more dense graph). If $\frac{{\mathcal {W}}_{\infty }}{{\mathcal {W}}_1} \approx 1$, then it means that the length of the critical path is very close to the total work (more sparse graph). Figure 14 shows the tightness for the proposed methods where we keep the value of ${\mathcal {W}}_1$ constant and vary the value of ${\mathcal {W}}_{\infty }$. The horizontal axis shows different $\frac{{\mathcal {W}}_{\infty }}{{\mathcal {W}}_1}$ ratios (significantly larger compared to the applications), and the vertical axis shows the tightness.

Initially, for both cases, the makespan increases since the critical path increases. Next, the tightness decreases as the $\frac{{\mathcal {W}}_{\infty }}{{\mathcal {W}}_1}$ increases since by increasing the ${\mathcal {W}}_{\infty }$, the impact of the heterogeneity increases.  has, on average, $6\%$ and at maximum $16\%$, less tight makespan compared to the exhaustive approach.

### Summary

From the simulation results, we can see that  provides tight makespan estimation compared to  and with low pessimism compared to the simulation of the execution $Sim$. We quantitatively verify the intuition by increasing the number of incompatible processors the makespan increases, which shows that the parallelism is restricted and leads to a larger estimation of the makespan. Next, we have seen that increasing the variation of the WCET across the different processor types leads to higher pessimism. Finally, by increasing the critical path’s workload and total workload ratio, the makespan increases because less parallelism is available.

## Comparison with similar approaches

This section compares our model with models in the literature that make more specific assumptions regarding multiprocessor platforms and applications. Initially, we compare our approach to approaches that assume the homogeneous and related multiprocessor model. Next, we compare our approach to a more specific application and platform model where Typed DAGs (Han et al. 2019) is assumed.

### Homogeneous and related multiprocessor models

Table 4 shows the specializations of the proposed formula to formulas proposed and used in related work. If $\delta ^{i}_{t} = 1$, for any task of the application and any processor, the multiprocessor platform is homogeneous. For the platform capacity and heterogeneity it holds that $S_{M}^{'}=M$ and $\lambda _{}^{'}=(M-1)$, respectively. As a result, the proposed formula becomes the same formula ($\frac{ {\mathcal {W}}_1+ \frac{(M-1)}{1}\cdot {\mathcal {W}}_{\infty }}{M}$) developed in Graham (1969), Brent (1974) and used extensively in previous works, for example Blumofe and Leiserson (1999), Melani et al. (2015). Similarly, by assuming the same speeds for the processors for all the tasks, the formula is the same as the formula proposed in the context of the related multiprocessor model by Jiang et al. (2017).

**Table 4:** Specializations of the (U)nrelate multiprocessor model to (H)omegeneous, (R)elated multiprocessor models

	U This work	H Graham (1969)	R Jiang et al. (2017)
Heterogeneity	$\lambda _{}^{'}$	$M-1$	$\lambda _{}^{R} = \lambda _{}^{'}$
Capacity	$S_{M}^{'}$	$M$	$S_{M}^{R} = S_{M}^{'}$
Makespan	$\frac{{\mathcal {W}}_1+ \lambda _{}^{'} \cdot {\mathcal {W}}_{\infty }}{ S_{M}^{'} }$	$\frac{ {\mathcal {W}}_1+ \frac{(M-1)}{1} \cdot {\mathcal {W}}_{\infty }}{M}$	$\frac{{\mathcal {W}}_1+ \lambda _{}^{R} \cdot {\mathcal {W}}_{\infty }}{ S_{M}^{R} }$

### Typed DAG application model

In the work of Han et al. (2019), typed DAGs (i.e., every task is compatible with one processor type) are assumed, and two bounds are proposed to estimate the makespan. The proposed bounds strictly dominate the used baseline in Jaffe (1980) and, through simulation, outperforms the work by Yang et al. (2016) significantly. The first approach (NEW-B-1) is a generalization of the Graham (1969) for typed DAGs. The second bound (NEW-B-2) explores the structure of the DAG and provides a tighter makespan.

By restricting the compatibility of the tasks, the parallelism is reduced because fewer processors are available to execute every task. Also, the critical path may be spread to different processor types. As a result, all the nodes that belong to processor type $t_1$ can interfere with tasks that belong to the critical path and are executed on processor type $t_2$. The proposed analysis (Jaffe 1980; Han et al. 2019) reflects this problem by assuming no parallelism between tasks executed on different processor types. It sums, i.e., serializes, the execution of the nodes that belong to different types. This assumption limits parallelism significantly. For example, for any number of processors that a multiprocessor has, if there is only one processor of each type, all the approaches are equal to the sequential execution.

**Table 5:** Specializations of the unrelated to the typed-DAG application model

	This work	This work	NEW-B-1 Han et al. (2019)
Makespan			

We can model Typed DAGs with our settings by assuming for a task $\tau ^{i}$ that $\delta ^{i}_{t} = 1$ for the compatible processors and $\delta ^{i}_{t} = 0$ for the non-compatible processors. Let  denote the number of processors of type $t$ and let  be the minimum number of processors between the different processor types. Table 5 presents the calculations for the typed DAG model. By trivially applying the  approach, for any typed DAG the platform capacity is  and heterogeneity is $\lambda _{}^{'}= M-1$. So, the makespan of  is more pessimistic than Jaffe (1980) and, as a result, also than the (NEW-B-1) and (NEW-B-2) from Han et al. (2019). To reduce the pessimism, we can find the makespan by computing the capacity and the heterogeneity from the same permutation. More precisely, first, we compute the makespan for all the possible permutations. Then we find the maximum makespan among all the permutations, denoted as , and the platform capacity is  and heterogeneity is . This approach has exponential time complexity since all the permutations need to be searched. The  is better than  but still more pessimistic than (NEW-B-1) and (NEW-B-2). By assuming that the mapping of the tasks is known, we can calculate ${\mathcal {W}}_{\infty }^{t}$ and ${\mathcal {W}}_1^{t}$ for each . By serializing the execution between the types and applying our formula, we find the same formula as for (NEW-B-1), which is more pessimistic than (NEW-B-2).

## Related work

In Graham (1969), Brent (1974), a makespan calculation is presented for parallel applications modeled as DAGs executed on homogeneous multiprocessors. The work in Blumofe and Leiserson (1999) extends this bound in the Cilk programming model context. The Cilk-based parallel applications are modeled with a restricted version of DAGs, and the bound is extended to cover the work-stealing scheduler. In Voudouris et al. (2017), Chen et al. (2019) a formally proven timing anomaly-free dynamic scheduler is introduced that provides tighter and more scalable, for the number of tasks and number of processors, makespan estimations. The results of Voudouris et al. (2017), Chen et al. (2019) cannot be trivially applied to unrelated multiprocessors because the DAG can have different schedule lengths depending on the task-processor mapping.

In Bender and Rabin (2000), the scheduler of Cilk (Blumofe and Leiserson 1999) is adapted for related heterogeneous systems. They provide a makespan calculation methodology for Cilk-based applications that can be modeled as DAGs, and a makespan is introduced. Our approach considers the unrelated multiprocessor model, which is a more general model for the underlying platform.

The work in Sih and Lee (1993), Topcuoglu et al. (2002) considers *static* scheduling of applications modeled as DAGs on unrelated multiprocessor platforms, and the goal is to minimize the schedule length. An extensive comparison of different heuristics for static scheduling on heterogeneous systems can be found in Braun et al. (2001). In contrast, our approach considers *dynamic* scheduling that can utilize the platform more efficiently to achieve load balance among the processors.

In Lawler and Labetoulle (1978), global scheduling of independent tasks for unrelated multiprocessor scheduling is formulated and solved as an integer linear problem. In Andersson et al. (2010), Raravi et al. (2013) the problem of scheduling independent tasks on two types of unrelated heterogeneous multiprocessor platforms is considered, and further extension of the works in Andersson et al. (2010), Raravi et al. (2013) can be found in Raravi (2014). Next, Andersson and Raravi (2014) assumes implicit-deadline, independent tasks, unrelated multiprocessor platforms, and shared resources, a speed-up bound of $4 \cdot (1 + \epsilon )$, where $\epsilon$ depends on the number of shared resources and the resource requests from the tasks. The assumption of shared resources enriches the applicability of the model. However, we do not address this problem in this paper, and we leave it as future work. Furthermore, the work in Andersson and Raravi (2016) assumes constrained-deadline independent tasks and unrelated platforms but is limited to two processor types. The problem is formulated as an ILP, and a speed-up bound of 5 is guaranteed. Next, Baruah et al. (2019) with the ILP approach for constrained deadlines, independent tasks, unrelated multiprocessors, and partitioned scheduling, a speed-up bound of 7.83 is achieved. Our approach considers a more general application model which can exploit the parallelism that exists in the applications. In this work, we assume a single DAG, and our goal is to find the makespan which is needed for the analysis for the recurrent execution of DAGs.

Previous work for general-purpose scheduling on unrelated multiprocessors has focused on special cases of our system model either by limiting the structure of the DAG (Kumar et al. 2009) or by limiting the execution time of the tasks and their compatibility to the processors (Page 2019). In this work, we consider DAGs where each task can execute on any processor and can have any execution time. In addition, the proposed makespan can be applied to any priority ordering of the task that has the work-conserving and the greediness property. As a result, in this work, instead of focusing on finding a carefully optimized scheduler for special cases of the application or platform models, we opt to find a makespan computation formula that is general and has broad applicability.

The estimation of the makespan of a single DAG gives us the tool to analyze multiple DAGs with the sporadic DAG model (Baruah et al. 2015a, 2012). In Li et al. (2014), Melani et al. (2015), Pathan et al. (2018) global scheduling is assumed and analyzed for homogeneous multiprocessors. Furthermore, federated scheduling, which can be seen as a generalization of partitioned scheduling, recently has gained attention, and many recent works focus on this topic (Li et al. 2014; Jiang et al. 2017; Bhuiyan et al. 2018; Ueter et al. 2018). This paper proposes a single DAG analysis on unrelated multiprocessors, which is the first step towards the analysis of sporadic DAGs.

## Conclusion

We propose two approaches to calculate the upper bound on the worst-case-schedule-length (makespan) for applications modeled as DAGs and executed on unrelated multiprocessors using any work-conserving scheduler. First, with an exhaustive approach, we show that Comb can safely establish an upper bound of the makespan. Still, its applicability is limited to small platforms and DAGs because it has exponential time complexity. We use Comb to build the Fast makespan that trades off the precision, i.e., tightness, of Comb to achieve polynomial time complexity.

To quantitatively evaluate the makespan of Fast, we model as DAGs four OpenMP task-based parallel applications and synthetic workloads. We compare Fast to Comb to determine the tightness. Based on the simulation results, the Fast approach finds the makespan nearly as tight as the Comb approach. Furthermore, we compare Fast with the simulation of the assumed scheduler that is a lower bound on the best-achievable makespan and we show that its estimation has low pessimism.

The main advantage of the proposed approach is its generality because it can be applied to a broad range of platforms, applications, and schedulers. The unrelated model is very expressive and can model many available platforms today using a wide range of processor types and specialized application accelerators. The DAG model is capable of capturing the behavior of many parallel applications. The scheduler is dynamic, so it can deal with a large number of fine-grain tasks that, for example, an OpenMP parallel application can have. The scheduler also supports arbitrary compatibility of the tasks to the processors. So, we can model accelerators that are designed to perform a limited set of operations more efficiently. Furthermore, the scheduler does not assume any scheduling policy, so our analysis can be applied to many well-known work-conserving schedulers from the related work. By fixing the WCET relation of the tasks to the processors, we show that the proposed makespan specializes (derives the same closed-form solution) to well-known bounds for homogeneous multiprocessors and recently developed related multiprocessors and typed DAGs.

The main limitation of the paper is the abstraction of the platform’s architectural details. We do not consider any shared resources between the task. However, in practice, many hardware components, for example, memory and interconnect, are shared. The use of shared resources significantly complicates the problem, and detailed timing analysis is needed to determine the interference of the tasks. We do not address the issue of shared resources in this paper. However, we expect the shared resource timing analysis to be orthogonal with our analysis and that it would increase the applicability of the model.

To the best of our knowledge, no related work covers the combination of assumptions: DAG application model, unrelated multiprocessor model, and work-conserving scheduling. As future work, we plan to develop the analysis of multiple DAGs that are executed on unrelated multiprocessors with the use of the sporadic DAG model (Baruah et al. 2015a, 2012)

## Proofs of Lemmas 3 and 4

This Section contains the proofs of lemmas that are used for the proof of the efficient makespan calculation () given by Theorem (1).

### Proof of Lemma 3

Lemma 3 states that the platform capacity $S_{M}^{'}$ is always less or equal to minimum platform capacity between the different permutations $S_{M}^{}$. The proof of Lemma (3) is given as follows:

#### Proof

Let $\pi _{}^{}(x)$ denotes the $x^{th}$ task that belongs to a permutation $\pi _{}^{}$. For an arbitrary task $\pi _{}^{}(x)$ that it is executing on its $x^{th}$ fastest processor it holds that:

$$\begin{aligned} \mathcal {O}^{\pi _{}^{}(x)}_{x} \ge \min _{i=1}^{N} \{ \mathcal {O}^{i}_{x} \} \end{aligned}$$

Since the size of the $\mathcal {O}^{i}_{}$ is equal to the number of processors $M$ it holds that:

$$\begin{aligned} \sum _{x=1}^{M} \{ \mathcal {O}^{\pi _{}^{}(x)}_{x} \} \ge \sum _{x=1}^{M} \min _{i=1}^{N~} \{ \mathcal {O}^{i}_{x} \} \\ \end{aligned}$$

Since it holds for an arbitrary permutation $\pi _{}^{}$, it also holds for the permutation that provides the minimum value.

$$\begin{aligned} \min _{\pi _{}^{} \in \sigma _{M}} \left\{ \sum _{x=1}^{M} \mathcal {O}^{\pi _{}^{}(x)}_{x} \right\} \ge \sum _{x=1}^{M} \min _{i=1}^{N} \left\{ \mathcal {O}^{i}_{x} \right\} \end{aligned}$$

From the definitions of $S_{M}^{}$ and $S_{M}^{'}$ given by Eqs. (10) and (11) respectively, the statement of the lemma holds. $\square$

### Proof of Lemma 4

Lemma 4 states that the heterogeneity $\lambda _{}^{'}$ is always greater or equal to the maximum heterogeneity $\lambda _{}^{}$ between the different permutations. The proof of Lemma 4 follows.

#### Proof

Let $\overline{\mathcal {O}}_{x}^{\pi _{}^{}(x)}$  be the subset of speeds that are smaller than the $x^{th}$ fastest processor of the $x^{th}$ task that belongs to permutation $\pi _{}^{}$. For an arbitrary task $1 \le \pi _{}^{}(y) \le N$ that it is executed on its $y^{th}$ fastest processor, it holds that:

$$\begin{aligned} \mathcal {O}^{\pi _{}^{}(y)}_{y} \le \max _{j=1}^{N} \{ \mathcal {O}^{j}_{y} \} \end{aligned}$$

Equivalently,

$$\begin{aligned} \sum _{ y \in \overline{\mathcal {O}}_{x}^{\pi _{}^{}(x)}~ } \left\{ \mathcal {O}^{\pi _{}^{}(y)}_{y} \right\} \le \sum _{y \in \overline{\mathcal {O}}_{x}^{\pi _{}^{}(x)}~} \max _{j=1}^{N} \{ \mathcal {O}^{j}_{y} \} \end{aligned}$$

By dividing both sides with the speed of the $x^{th}$ fastest processor $\mathcal {O}^{\pi _{}^{}(x)}_{x} \ne 0$, of task $\pi _{}^{}(x)$ we have:

$$\begin{aligned} \frac{\sum _{ y \in \overline{\mathcal {O}}_{x}^{\pi _{}^{}(x)}~ } \{ \mathcal {O}^{\pi _{}^{}(y)}_{y} \}}{\mathcal {O}^{\pi _{}^{}(x)}_{x}} \le \frac{ \sum _{y \in \overline{\mathcal {O}}_{x}^{\pi _{}^{}(x)}~} \max _{j=1}^{N} \{ \mathcal {O}^{j}_{y} \} }{\mathcal {O}^{\pi _{}^{}(x)}_{x}} \end{aligned}$$

Since the inequality holds for any processor *x* it holds also for the processor that maximizes the two sides of the inequality:

$$\begin{aligned} \max _{x=1}^{M} \left\{ \frac{\sum _{ y \in \overline{\mathcal {O}}_{x}^{\pi _{}^{}(x)}~ } \{ \mathcal {O}^{\pi _{}^{}(y)}_{y} \}}{\mathcal {O}^{\pi _{}^{}(x)}_{x}} \right\} \le \max _{x=1}^{M} \left\{ \frac{ \sum _{y \in \overline{\mathcal {O}}_{x}^{\pi _{}^{}(x)}~} \max _{j=1}^{N} \{ \mathcal {O}^{j}_{y} \} }{\mathcal {O}^{\pi _{}^{}(y)}_{y}} \right\} \end{aligned}$$

Since it holds for an arbitrary task with index $\pi _{}^{}(x)$ that belongs to an arbitrary permutation $\pi _{}^{}$ it also holds for any task of the application $1 \le j \le N$ and we have:

26 $$\begin{aligned} \max _{x=1}^{M} \left\{ \frac{ \sum _{y \in \overline{\mathcal {O}}_{x}^{\pi _{}^{}(x)}~} \max _{j=1}^{N} \{ \mathcal {O}^{j}_{y} \} }{\mathcal {O}^{\pi _{}^{}(x)}_{x}} \right\} = \max _{x=1}^{M} \left\{ \frac{ \sum _{y \in \overline{\mathcal {O}}_{x}^{k}} \max _{j=1}^{N} \{\mathcal {O}^{j}_{y}\}}{\mathcal {O}^{k}_{x}}\right\} \end{aligned}$$

Since $1 \le k \le N~$ it also holds that:

27 $$\begin{aligned} \max _{x=1}^{M} \left\{ \frac{ \sum _{y \in \overline{\mathcal {O}}_{x}^{k}} \max _{j=1}^{N} \{ \mathcal {O}^{j}_{y} \} }{\mathcal {O}^{k}_{x}} \right\} \le \max _{i=1}^{N} \left\{ \max _{x=1}^{M} \{ \frac{ \sum _{y \in \overline{\mathcal {O}}_{x}^{i}} \max _{j=1}^{N} \{ \mathcal {O}^{j}_{y} \} }{\mathcal {O}^{i}_{x}} \} \right\} \end{aligned}$$

From (26) and (27) we have:

$$\begin{aligned} \max _{x=1}^{M} \left\{ \frac{ \sum _{y \in \overline{\mathcal {O}}_{x}^{\pi _{}^{}(x)}~} \max _{j=1}^{N} \{ \mathcal {O}^{j}_{y} \} }{\mathcal {O}^{\pi _{}^{}(x)}_{x}} \right\} \le \max _{i=1}^{N} \left\{ \max _{x=1}^{M} \{ \frac{ \sum _{y \in \overline{\mathcal {O}}_{x}^{i}} \max _{j=1}^{N} \{ \mathcal {O}^{j}_{y} \} }{\mathcal {O}^{i}_{x}}\}\right\} \end{aligned}$$

Let $\pi _{}^{\prime }$ be the permutation that provides the maximum value for the maximum accumulated capacity loss. Since it holds for any $\pi _{}^{}$ it also holds for the permutation $\pi _{}^{\prime }$, we have:

$$\begin{aligned} \max _{k=1}^{M} \left\{ \frac{\sum _{ y \in \overline{\mathcal {O}}_{x}^{\pi _{}^{'}(x)} } \{ \mathcal {O}^{\pi _{}^{'}(y)}_{y}\}}{\mathcal {O}^{\pi _{}^{'}(x)}_{x} } \right\} \le \max _{i=1}^{N} \left\{ \max _{x=1}^{M} \left\{ \frac{ \sum _{y \in \overline{\mathcal {O}}_{x}^{i}} \max _{j=1}^{N} \{ \mathcal {O}^{j}_{y} \} }{\mathcal {O}^{i}_{x}} \right\} \right\} \end{aligned}$$

Equivalently,

$$\begin{aligned} \max _{\pi _{}^{} \in \sigma _{M}} \left\{ \max _{k=1}^{M} \left\{ \frac{\sum _{ y \in \overline{\mathcal {O}}_{x}^{\pi _{}^{}(x)}~ } \{\mathcal {O}^{\pi _{}^{}(y)}_{y}\}}{\mathcal {O}^{\pi _{}^{}(x)}_{x}}\right\} \right\} \le \max _{i=1}^{N} \left\{ \max _{x=1}^{M} \left\{ \frac{ \sum _{y \in \overline{\mathcal {O}}_{x}^{i}} \max _{j=1}^{N} \{ \mathcal {O}^{j}_{y} \} }{\mathcal {O}^{i}_{x}}\right\} \right\} \end{aligned}$$

From the definitions of $\lambda _{}^{}$, $\lambda _{}^{'}$ and $idle^{i}_{x}$ given by Eqs. (13), (15) and (14), we have: $\lambda _{}^{} \le \lambda _{}^{'}$. As a result $\lambda _{}^{'}$ is always greater than or equal to $\lambda _{}^{}$. $\square$

## Footnotes

1 Unlike unrelated heterogeneous multiprocessors, related heterogeneous multiprocessors (also known as uniform multiprocessors (Baruah et al. 2015a)) have a specific speed for each processor type and all the tasks execute at that specific speed on any processor of that particular processor type. The homogeneous multiprocessor model has exactly one processor type, and all the tasks execute at the same speed on *all* processors.

2 The asterisk is used as a wildcard task executing at its preferred processor.

## References

[CR1] Andersson B, Raravi G (2014). Real-time scheduling with resource sharing on heterogeneous multiprocessors. Real-time systems.

[CR2] Andersson B, Raravi G (2016) Scheduling constrained-deadline parallel tasks on two-type heterogeneous multiprocessors. In: Proceedings of the 24th International Conference on Real-Time Networks and Systems, ACM

[CR3] Andersson B, Raravi G, Bletsas K (2010) Assigning real-time tasks on heterogeneous multiprocessors with two unrelated types of processors. In: IEEE RTSS

[CR4] ARM (2011) big.little technology: the future of mobile. White paper

[CR5] Baruah S, Bertogna M, Buttazzo G (2015). Multiprocessor scheduling for real-time systems.

[CR6] Baruah SK, Bonifaci V, Bruni R, Marchetti-Spaccamela A (2019). Ilp models for the allocation of recurrent workloads upon heterogeneous multiprocessors. J Scheduling.

[CR7] Baruah S, Bonifaci V, Marchetti-Spaccamela A (2015b) The global edf scheduling of systems of conditional sporadic dag tasks. In: IEEE ECRTS

[CR8] Baruah S, Bonifaci V, Marchetti-Spaccamela A, Stougie L, Wiese A (2012) A generalized parallel task model for recurrent real-time processes. In: IEEE RTSS

[CR9] Bender MA, Rabin MO (2000) Scheduling cilk multithreaded parallel programs on processors of different speeds. In: ACM SPAA

[CR10] Bhuiyan A, Guo Z, Saifullah A, Guan N, Xiong H (2018) Energy-efficient real-time scheduling of dag tasks. In: ACM TECS

[CR11] Blumofe RD, Leiserson CE (1999). Scheduling multithreaded computations by work stealing. J ACM.

[CR12] Braun TD (2001). A comparison of eleven static heuristics for mapping a class of independent tasks onto heterogeneous distributed computing systems. J Parallel Distrib Comput.

[CR13] Brent RP (1974). The parallel evaluation of general arithmetic expressions. J ACM.

[CR14] Chen P, Liu W, Jiang X, He Q, Guan N (2019) Timing-anomaly free dynamic scheduling of conditional dag tasks on multi-core systems. In: ACM Transactions on Embedded Computing Systems (TECS)

[CR15] Chronaki K, et al. (2015) Criticality-aware dynamic task scheduling for heterogeneous architectures. In: ACM, ICS

[CR16] Chwa HS, Seo J, Lee J, Shin I (2015) Optimal real-time scheduling on two-type heterogeneous multicore platforms. In: IEEE RTSS

[CR17] Duran A, et al. (2002) Barcelona openmp tasks suite: a set of benchmarks targeting the exploitation of task parallelism in openmp. In: ICPP

[CR18] Esmaeilzadeh H, Blem E, Amant RS, Sankaralingam K, Burger D (2011) Dark silicon and the end of multicore scaling. In: IEEE ISCA

[CR19] Funk S, Goossens J, Baruah S (2001) On-line scheduling on uniform multiprocessors. In: IEEE RTSS

[CR20] Garey MR, Johnson DS (2002). Computers and intractability.

[CR21] Graham RL (1969). Bounds on multiprocessing timing anomalies. SIAM J Appl Math.

[CR22] Gupta A, Im S, Krishnaswamy R, Moseley B, Pruhs K (2012) Scheduling heterogeneous processors isn’t as easy as you think. In: ACM-SIAM SODA

[CR23] Han M, Guan N, Sun J, He Q, Deng Q, Liu W (2019) Response time bounds for typed dag parallel tasks on heterogeneous multi-cores. In: IEEE TPDS

[CR24] Jaffe JM (1980). Bounds on the scheduling of typed task systems. SIAM J Comput.

[CR25] Jiang X, Guan N, Long X, Yi W (2017) Semi-federated scheduling of parallel real-time tasks on multiprocessors. In: IEEE RTSS

[CR26] Kumar VA, Marathe MV, Parthasarathy S, Srinivasan A (2009). Scheduling on unrelated machines under tree-like precedence constraints. Algorithmica.

[CR27] Lakshmanan K, Kato S, Rajkumar R (2010) Scheduling parallel real-time tasks on multi-core processors. In: IEEE RTSS

[CR28] Lawler EL, Labetoulle J (1978). On preemptive scheduling of unrelated parallel processors by linear programming. J ACM (JACM).

[CR29] Li J, Chen JJ, Agrawal K, Lu C, Gill C, Saifullah A (2014) Analysis of federated and global scheduling for parallel real-time tasks. In: IEEE ECRTS

[CR30] Melani A, Bertogna M, Bonifaci V, Marchetti-Spaccamela A, Buttazzo GC (2015) Response-time analysis of conditional dag tasks in multiprocessor systems. In: ECRTS

[CR31] Page DR (2019) Approximation algorithms for problems in makespan minimization on unrelated parallel machines. The University of Western Ontario (PhD thesis)

[CR32] Pathan R, Voudouris P, Stenström P (2018) Scheduling parallel real-time recurrent tasks on multicore platforms. In: IEEE TPDS

[CR33] Peter Greenhalgh A (2011) Big.little processing with arm cortex-a15 and cortex-a7 improving energy efficiency in high-performance mobile platforms. White paper, http://www.cl.cam.ac.uk/~rdm34/big.LITTLE.pdf

[CR34] Raravi G (2014) Real-time scheduling on heterogeneous multiprocessors. Faculty of Engineering, University of Porto (PhD thesis)

[CR35] Raravi G, Andersson B, Bletsas K (2013). Assigning real-time tasks on heterogeneous multiprocessors with two unrelated types of processors.

[CR36] Sih GC, Lee EA (1993) A compile-time scheduling heuristic for interconnection-constrained heterogeneous processor architectures. In: IEEE TPDS

[CR37] Topcuoglu H, Hariri S, My Wu (2002) Performance-effective and low-complexity task scheduling for heterogeneous computing. In: IEEE TPDS

[CR38] Ueter N, von der Brüggen G, Chen JJ, Li J, Agrawal K (2018) Reservation-based federated scheduling for parallel real-time tasks. In: IEEE RTSS

[CR39] Voudouris P, Stenström P, Pathan R (2017) Timing-anomaly free dynamic scheduling of task-based parallel applications. In: IEEE RTAS

[CR40] West DB (2001). Introduction to graph theory.

[CR41] Yang K, Yang M, Anderson JH (2016) Reducing response-time bounds for dag-based task systems on heterogeneous multicore platforms. In: ACM RTNS

